# Endoplasmic reticulum-to-Golgi transitions upon herpes virus infection

**DOI:** 10.12688/f1000research.12252.2

**Published:** 2018-02-28

**Authors:** Peter Wild, Andres Kaech, Elisabeth M. Schraner, Ladina Walser, Mathias Ackermann

**Affiliations:** 1 Institute of Veterinary Anatomy, Zürich, Switzerland; 2 Institute of Virology, Zürich, Switzerland; 3 Center for Microscopy and Image Analysis, Zürich, Switzerland

**Keywords:** herpes virus, envelopment, egress pathway, endoplasmic reticulum, Golgi complex, intraluminal transport, brefeldin A

## Abstract

**Background**: Herpesvirus capsids are assembled in the nucleus, translocated to the perinuclear space by budding, acquiring tegument and envelope, or released to the cytoplasm via impaired nuclear envelope. One model proposes that envelopment, “de-envelopment” and “re-envelopment” is essential for production of infectious virus. Glycoproteins gB/gH were reported to be essential for de-envelopment, by fusion of the “primary” envelope with the outer nuclear membrane. Yet, a high proportion of enveloped virions generated from genomes with deleted gB/gH were found in the cytoplasm and extracellular space, suggesting the existence of alternative exit routes.

**Methods**: We investigated the relatedness between the nuclear envelope and membranes of the endoplasmic reticulum and Golgi complex, in cells infected with either herpes simplex virus 1 (HSV-1) or a Us3 deletion mutant thereof, or with bovine herpesvirus 1 (BoHV-1) by transmission and scanning electron microscopy, employing freezing technique protocols.

**Results**:  The Golgi complex is a compact entity in a juxtanuclear position covered by a membrane on the *cis* face. Golgi membranes merge with membranes of the endoplasmic reticulum forming an entity with the perinuclear space. All compartments contained enveloped virions. After treatment with brefeldin A, HSV-1 virions aggregated in the perinuclear space and endoplasmic reticulum, while infectious progeny virus was still produced.

**Conclusions**: The data suggest that virions derived by budding at nuclear membranes are intraluminally transported from the perinuclear space via Golgi -endoplasmic reticulum transitions into Golgi cisternae for packaging. Virions derived by budding at nuclear membranes are infective like Us3 deletion mutants, which  accumulate in the perinuclear space. Therefore, i) de-envelopment followed by re-envelopment is not essential for production of infective progeny virus, ii) the process taking place at the outer nuclear membrane is budding not fusion, and iii) naked capsids gain access to the cytoplasmic matrix via impaired nuclear envelope as reported earlier.

## Introduction

The Golgi complex plays a crucial role in the secretory pathway. Cargo is transported from the endoplasmic reticulum (ER) to the Golgi complex via vesicles that derive from ER exit sites ( Bonifacino & Glick, 2004). Tubules are involved in anterograde as well as in retrograde transport ( Lippincott-Schwartz *et al.*, 1990; Lippincott-Schwartz *et al.*, 1989). The sole paradigm of vesicular transport between ER and Golgi may evolve to account for the results of new technologies ( Lippincott-Schwartz, 2011). Indeed, cargo may also be transported through an ER-Golgi intermediate compartment (ERGIC) ( Hauri & Schweizer, 1992; Klumperman, 2000; Saraste *et al.*, 2009). Whether the ERGIC is a stable structure, is under debate ( Ben-Tekaya *et al.*, 2005). There are also transitional elements connecting Golgi membranes to ER membranes ( Pavelka & Roth, 2015; Polishchuk & Mironov, 2004; Vivero-Salmerón *et al.*, 2008) possibly enabling direct transportation of cargo from ER cisternae into Golgi cisternae. Once in the Golgi cisternae, cargo is packaged into secretory granules for exocytotic release ( Palade, 1975). Packaging of cargo is accompanied by loss of Golgi membranes. To maintain Golgi structure and function, multiple recycling processes take place ( Orci *et al.*, 1981). Although the structure and function of the Golgi complex has been investigated for decades, many uncertainties remain e.g. Golgi maturation and functionality of the *trans* Golgi network (TGN) ( Emr *et al.*, 2009).

The Golgi complex plays also a crucial role in herpes virus morphogenesis and intracellular transport ( Roizman *et al.*, 2014). Herpes viruses comprise the capsid, tegument and envelope, with embedded glycoproteins. Capsids are assembled in nuclei of host cells and transported to the Golgi complex concomitantly acquiring the envelope and tegument. Yet, this transport is not fully understood and controversially discussed. For certain, capsids bud at the inner nuclear membrane (INM) acquiring an envelope and tegument. Since the discovery of capsids in the cytoplasm adjacent to the nucleus ( Stackpole, 1969) the virions are believed to be released from the PNS via de-enveloped by fusion of the viral envelope with the outer nuclear membrane (ONM) or with adjacent ER membranes releasing capsid and tegument into the cytoplasmic matrix. These capsids then are re-enveloped by budding at membranes of the TGN ( Mettenleiter *et al.*, 2013) acquiring again an envelope and tegument. Concomitantly, a small concentric transport vacuole is formed enclosing the enveloped virion. This process is referred to as wrapping ( Roizman *et al.*, 2014). Alternatively, capsids may be enveloped at endosomes in cells infected with HSV-1 ( Albecka *et al.*, 2016; Hollinshead *et al.*, 2012) or varicella-zoster virus (VZV) ( Buckingham *et al.,* 2016; Grose *et al.,* 2016). Virions have been repeatedly shown in ER cisternae suggesting that virions are intraluminally transported ( Gilbert *et al.*, 1994; Granzow *et al.*, 1997; ( Harson & Grose, 1995) Radsak *et al.*, 1996; Schwartz & Roizman, 1969; Stannard *et al.*, 1996; Sutter *et al.*, 2012; Whealy *et al.*, 1991; Wild *et al.*, 2002) into ER cisternae, whose membranes are connected to the ONM. Virions may be released from the PNS and ER by formation of vesicles engulfing virions ( Campadelli-Fiume *et al.,* 1991; Epstein, 1962; Harson & Grose, 1995; Torrisi *et al.,* 1992; Torrisi *et al.,* 1999). Alternatively, virions were suggested to be farther transported via ER-Golgi transitions into Golgi cisternae ( Leuzinger *et al.*, 2005; Wild *et al.*, 2015; Wild *et al.*, 2002). Importantly, intraluminal transportation requires mechanisms for preventing the viral envelope from fusion with the membrane the virions are transported along. Thus, virions can either be intraluminally transported or the viral envelope fuses with the ONM or ER membranes. There is no doubt that virions can be intraluminally transported and to aggregate in the PNS-ER compartment, e.g. in the absence of the Us3 kinase, raising the question how capsids gain access to the cytoplasmic matrix.

The question remains how naked capsids gain access to the cytoplasmic matrix. It has been clearly shown that nuclear pores dilate leading to impairment of the nuclear envelope, through which capsids are released from the nuclear periphery into the cytoplasmic matrix ( Leuzinger *et al.*, 2005; Wild *et al.*, 2005; Wild *et al.*, 2009) or disrupted nuclear membranes ( Borchers & Oezel, 1993; Klupp *et al.*, 2011). Notably, pore impairment is the initial step in breakdown of the nuclear envelope ( Terasaki *et al.*, 2001) that takes place when HSV-1 infection proceeds ( Maric *et al.*, 2014). The capsids in the cytoplasmic matrix are transported to any site of the Golgi complex ( Wild *et al.*, 2002) and are enveloped either by wrapping (see above) or by budding into Golgi cisternae and/or vacuoles, which may enlarge to engulf multiple virions ( Homman-Loudiyi *et al.*, 2003; Leuzinger *et al.*, 2005; Stannard *et al.*, 1996; Sutter *et al.*, 2012; Wild *et al.*, 2015; Wild *et al.*, 2002). Capsids bud, though less frequently, also at the ONM and RER membranes ( Leuzinger *et al.*, 2005; Wild *et al.*, 2005), and are intraluminally transported to a yet unknown destination.

Although the phenotypes of the capsid transport across the ONM exhibit all characteristics of budding ( Bonifacino & Glick, 2004; Harrison, 2015; Jahn *et al.*, 2003; Kanaseki *et al.*, 1997; Lee, 2010; Mayer, 2002) the de-envelopment theory is still favored. Fusion of the viral envelope with the ONM requires the glycoproteins gB and gH ( Farnsworth *et al.*, 2007). Nonetheless, various stages of capsid transportation across the ONM and ER membranes in the absence of the glycoproteins gB/gH have been shown (see Figure 2 in Farnsworth *et al.,* 2007). Furthermore, a substantial proportion of virions lacking gB/gH were reported to be in the cytoplasm and extracellular space. These two facts strongly suggest another pathway of virus particles out of the PNS. The Us3 kinase is involved in nucleus to cytoplasm capsid translocation ( Poon *et al.,* 2006) via phosphorylation of viral proteins including gB ( Wisner *et al.,* 2009), UL31 and UL34 ( Mou *et al.,* 2009; Ryckman & Roller, 2004). In the absence of the Us3 kinase, 98% of all produced virions accumulate in the PNS-ER compartment ( Wild *et al.*, 2015). These virions are infective ( Reynolds *et al.*, 2002; Wisner *et al.*, 2009). Us3 is also involved in downregulation of phospholipid biosynthesis ( Wild *et al.,* 2012) induced by HSV-1 ( Sutter *et al.,* 2012), and in blocking of apoptosis ( Wang *et al.,* 2011).

Transportation of virions out of the PNS into ER raises the question of their destination. As stated above, the viral envelope cannot fuse because transportation of a membrane bound particle along membranes needs protection from fusion. A possible destination of intraluminal transported virions could be further transportation into Golgi cisternae via ER-to-Golgi transitions as suggested by ( Leuzinger *et al.*, 2005). ER-to-Golgi transitions have been shown in many cells since the early 1970s ( Claude, 1970). Therefore, we investigated cells infected with BoHV-1, a member of the subfamily varicella virus, or with HSV-1, a member of the subfamily simplex virus, or a Us3 deletion mutant thereof, because the Golgi complex enlarges in the absence of Us3 ( Wild *et al.,* 2012) by cryo-field emission scanning electron microscopy (Cryo-FESEM) and transmission electron microscopy (TEM), employing protocols for improved spatial and temporal resolution ( Ellinger *et al.*, 2010; Mueller, 1992). We show that the Golgi complex is a tightly packed organelle, that ER-to-Golgi transitions are present, and that virions aggregate within the ER after exposure cells to brefeldin A (BFA), which disintegrates the Golgi complex within minutes ( Hess *et al.*, 2000), suggesting an intraluminal transportation route. Moreover, we observe that intraluminal virions are densely coated with a proteinaceous layer that arises during budding at nuclear membranes. Therefore, we propose that the significance of the dense coat is protecting the viral envelope from fusion with the membranes along which virions are transported, in a similar manner as clathrin protects coated vesicles from fusion.

## Materials and methods

### Cells and viruses

Vero cells and MDBK cells (European Collection of Cell Cultures) were grown in Dulbecco’s modified minimal essential medium (DMEM; Gibco, Bethesda, MD, USA) supplemented with penicillin (100 U/ml), streptomycin (100 μg/ml) and 10% fetal bovine serum (FBS; Gibco). The Us3 deletion mutant R7041(ΔUs3) and the repair mutant R2641 ( Longnecker & Roizman, 1987; Purves *et al.*, 1987) were kindly provided by Bernard Roizman (The Marjorie B. Kovler Viral Oncology Laboratories, University of Chicago, Illinois, USA). Wild-type herpes simplex virus 1 (wt HSV-1) strain F (Ejercito *et al.*, J. Gen. Virol. 2:357–364, 1968), R7041(ΔUs3) and R2641 were propagated in Vero cells, and bovine herpes virus 1 (BoHV-1: Metzler *et al.*, Arch. Virol. 87: 205–217, 1986) in MDBK cells. Virus yields were determined by plaque titration.

### Cryo-fixation for transmission electron microscopy

50 μm thick sapphire disks (Bruegger, Minusio, Switzerland) measuring 3 mm in diameter were coated with 8–10 nm carbon, obtained by evaporation under high vacuum conditions to enhance cell growth and to facilitate detachment of cells from the sapphire disks after embedding. Vero and MDBK cells were grown for 2 days on sapphire disks placed in 6 well plates. Cells were inoculated with R7041(ΔUs3), the repair mutant R2641, wt HSV-1 or BoHV-1 at a MOI of 5, incubated at 37°C, and fixed at 8 to 20 hpi by adding 0.25% glutaraldehyde to the medium prior to freezing in a high-pressure freezing unit (HPM010; Science Services, Munich, Germany) and processed as described in detail ( Wild, 2008). In brief, the frozen water was substituted with acetone in a freeze-substitution unit (FS 7500; Boeckeler Instruments, Tucson, AZ, USA) at -88°C with acetone and subsequently fixed with 0.25% glutaraldehyde and 0.5% osmium tetroxide raising the temperature gradually to +2°C to achieve good contrast of membranes ( Wild *et al.*, 2001), and embedded in epon at 4°C followed by polymerization at 60°C for 2.5 days. After removal of sapphire disks by immersion in liquid nitrogen, serial sections of 60 to 90 nm thickness were analyzed in a transmission electron microscope (CM12; FEI, Eindhoven, The Netherlands) equipped with a CCD camera (Ultrascan 1000; Gatan, Pleasanton, CA, USA) at an acceleration voltage of 100 kV.

### Cell exposure to brefeldin A

Cells grown on sapphire disks were inoculated with wt HSV-1 at a MOI of 5 and incubated at 37°C. Stock solution (5 mg BFA solved in 0.5 ml methanol) was diluted with medium 1:10. One µl/ml medium (1µg/ml) of this solution was added to cell cultures at 5, 8, 12 and 16 hpi. Cells were high-pressure frozen at indicated times, and prepared for TEM. To quantify virus phenotypes and their location, 10 images of cellular profiles were taken at random from ultrathin sections of monolayers exposed to BFA from 5, 8, 12 or 16 hpi to 20 hpi of 5 independent experiments. Capsids budding at the INM, ONM, ER and Golgi membranes, capsids undergoing wrapping, as wells as virions in the PNS, ER, Golgi cisternae and vacuoles were counted. The means expressed per cellular profile were compared applying a Student’s t-test using GraphPad Prism 3 software.

For determination of infectious progeny virus produced after BFA exposure, cells were grown in 10 ml Falcon flasks, inoculated with wt HSV-1 at a MOI of 5, and exposed to BFA from 5, 8, 12 or 16 h to 20 hpi. Cells were harvested at 5, 8, 12, 16 and 20 hpi for determination of infectious progeny virus by plaque titration. Since the Golgi complex reacts immediately to BFA by disintegration, infectious viruses produced after BFA exposure were considered to have derived by budding at membranes other than those of the Golgi complex.

### Cryo-Field Emission Scanning Electron Microscopy (Cryo-FESEM)

Vero cells were grown in 25 cm ^2^ cell culture flasks for 2 days prior to inoculation with wt HSV-1, R7041(ΔUs3) or R2641 at MOI of 5. Cells were harvested at 9 to 12 hpi by trypsinization followed by centrifugation at 150 x g for 8 min. Pellets were re-suspended in 1 ml fresh medium, collected in Eppendorf tubes and fixed by adding 0.25% glutaraldehyde to the medium. The suspension was kept in the tubes at 4°C until cells were sedimented. After removal of the supernatant, cells were frozen in a high-pressure freezing machine EM HPM100 (Leica Microsystems, Vienna, Austria) as described in detail previously ( Wild *et al.*, 2012; Wild *et al.*, 2009). Cells were fractured at -120°C in a freeze-fracturing device BAF 060 (Leica Microsystems) in a vacuum of 10 ^-7^ mbar. The fractured surfaces were partially freeze-dried (“etched”) at -105°C for 2 min, and coated with 2.5 nm platinum/carbon by electron beam evaporation at an angle of 45°. Some specimens were coated additionally with 4 nm of carbon to reduce electron beam damage during imaging at high magnifications. Specimens were imaged in an Auriga 40 Cross Beam system (Zeiss, Oberkochen, Germany) equipped with a cryo-stage (Leica Microsystems) at -115°C and an acceleration voltage of 5 kV using the inlens secondary electron detector.

### Confocal microscopy

Cells were grown for 2 days on 0.17 mm thick cover slips of 12 mm in diameter (Assistent, Sondheim, Germany) and inoculated with R7041(ΔUs3), wt HSV-1 or R2641 at a MOI of 5 and incubated at 37°C. After fixation with 2% formaldehyde for 25 min at room temperature, cells were briefly washed with PBS and stored in PBS at 4°C until further processing. Then, cells were permeabilized with 0.1% Triton-X-100 at room temperature for 7 min and blocked with 3% bovine serum albumin in PBS containing 0.05% Tween 20 (PBST). To identify the Golgi complex, cells were incubated with recombinant monoclonal antibodies against the *cis*-Golgi protein GM130, abcam EP892Y (Abcam, Cambridge, UK) at a dilution of 1:1000 for 2 h at room temperature followed by incubation with Alexa 647 (Molecular Probes, Eugene, OR, USA) as secondary antibodies, diluted 1:500, for 1 h at room temperature. For identification of the *trans*-Golgi, TGN46 (NBP1-49643, Novus Biologicals Europe, Abingdon, UK) was used at a dilution of 1:100, followed by Alexa 488 (Molecular Probes), diluted 1:500. To identify infected cells, antibodies against glycoprotein gB (Novus Biological, Littleton, CO, USA) diluted 1:250, and Alexa 594 (Molecular Probes) diluted 1:500, as secondary antibodies were used. After staining nuclei with 4',6-Diamidino-2-phenylindol (DAPI; Roche, Mannheim, Germany), cells were embedded in glycergel mounting media (Dako North America, Carpinteria, CA, USA) and 25 mg/ml 1,4-diazabicyclo [2.2.2] octane (DABCO; Fluka, Buchs, Switzerland). Specimens were analyzed using a confocal laser scanning microscope (SP2; Leica, Wetzlar, Germany). Images were deconvolved employing the deconvolution algorithm of the program suite Huygens Essential (SVI, Hilversum, The Netherlands).

## Results

### The Golgi complex is a tightly packed entity situated close to the nucleus

**Figure 1.**
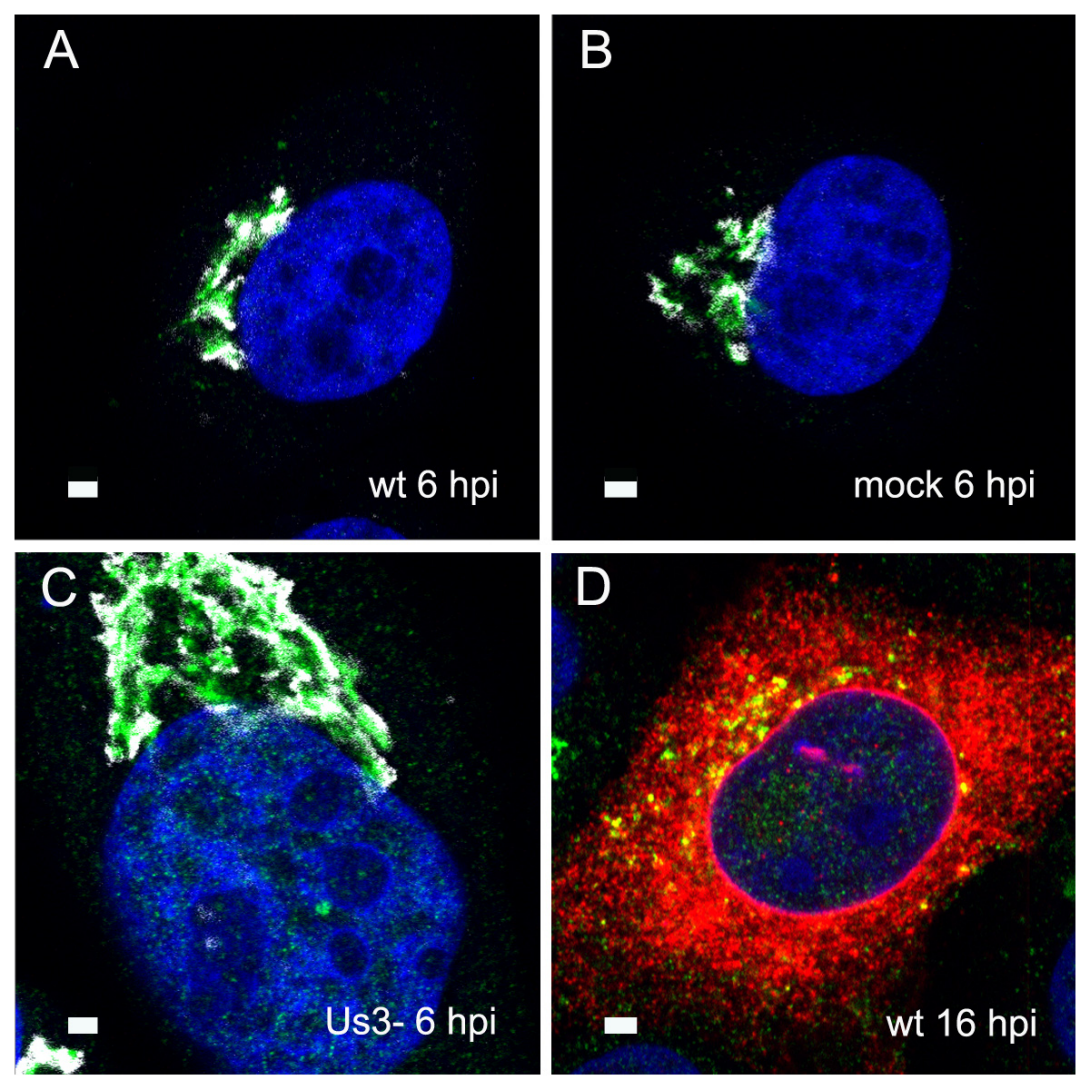
Immunolabelling of the Golgi complex. Confocal microscopy of the *trans*-face (green) and the *cis*-face (white) of Vero cells, immunolabeled with anti TGN46 antibodies (green) and anti GM130 antibodies (white) at 6 h after inoculation with HSV-1 ( **A**), R7041(ΔUs3) ( **C**) after mock infection ( **B**), as well a at 16 hpi with wt HSV-1 ( **D**) together with immunolabeling of the viral glycoprotein gB (red). The Golgi complex was always in a juxtanuclear position and enormously enlarged after R7041(ΔUs3). Note the enlargement of the nucleus after infection with R7041(ΔUs3). Bars 1 µm.

**Figure 2.**
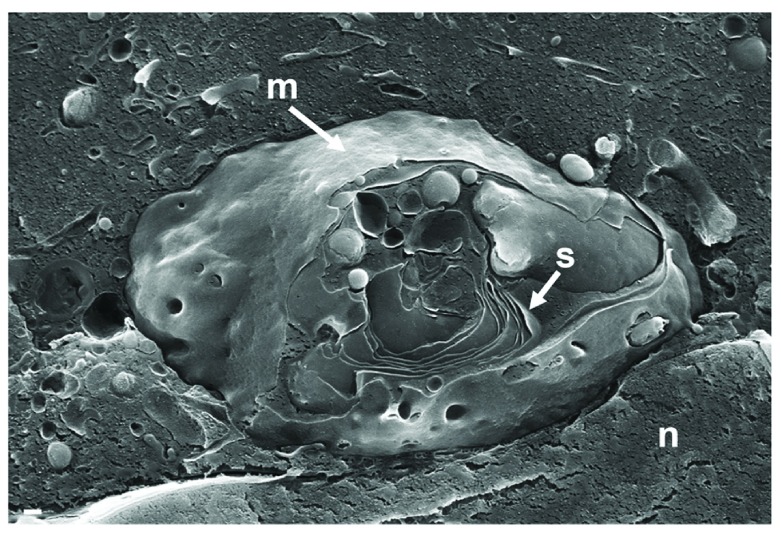
Cryo-FESEM of a Golgi complex in close vicinity to the nucleus (n) in a Vero cell, at 10 hpi with wt HSV-1. The entire visible surface is covered by an intact membrane (m) except at the part it is broken away giving view to Golgi stacks (s). Bars: 200 nm.

**Figure 3.**
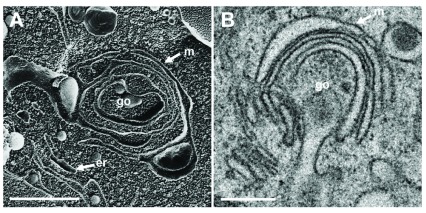
Golgi fields not involved in virus maturation and transport. The Golgi complex, as revealed in freeze-fracture planes ( **A**) and in thin sections ( **B**), is entirely covered at the *cis*-face by the membrane (m) of the outermost cisterna. Bars: 100 nm.

To localize the Golgi complex, we first imaged the Golgi complex by confocal microscopy after labeling the *cis*-face with anti GM 130 antibodies, and the *trans*-face with anti TGN46 antibodies in Vero cells. The Golgi complex was always found close to the nucleus in HSV-1, R7041(ΔUs3), or mock infected cell ( Figure 1). At 6 hpi with wt HSV-1, the trans-Golgi was of similar size and shape as in mock infected cells whereas the *cis*-Golgi had disappeared by 16 hpi, and the *trans*-Golgi became fragmented. In contrast, the Golgi complex had enormously enlarged after infection with the Us3 deletion mutant R7041(ΔUs3), which is in line with the enlargement of the surface area of Golgi membranes as revealed by electron microscopic morphometry ( Wild *et al.,* 2012), and reflects the significance of Us3 in downregulation of phospholipid synthesis induced by HSV-1 ( Sutter *et al.,* 2012). Next, high resolution cryo-electron microscopy of freeze fractured cells demonstrated the bell-shaped form of the Golgi complex, and its localization close to the nucleus ( Figure 2). This image also shows that the Golgi complex is a complex tightly packed entity with a diameter of approximately 6 µm. The Golgi complex is separated from the cytoplasmic matrix by an intact membrane covering the whole visible surface of the *cis*-face. The large dimension makes clear that the detected ultrastructural details depend on a large scale on how and where the Golgi complex is hit in a given section plane for studying by TEM. Both freeze-fracture planes and thin sections of central regions show that the membrane of the outermost cisterna covers the *cis*-side ( Figure 3).

### ER-to-Golgi transitions

**Figure 4.**
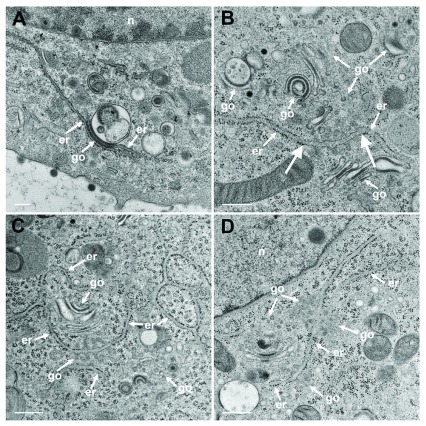
TEM of R7041(ΔUs3) infected Vero cells at 12 hpi. ( **A**) The ER (er) runs from the nuclear (n) periphery towards a Golgi field (go), continuing into the membrane of the outermost stack and further into the cytoplasmic matrix. The membranes of the second stack continue also into ER membranes. ( **B**) An ER cisterna runs through multiple small Golgi fields, whereby the ER membranes turn into Golgi membranes (thick arrows). ( **C**) ER membranes forming a network continue into Golgi membranes. ( **D**) ER membranes run through two Golgi fields, turning each time into Golgi membranes. Bars: 500 nm.

**Figure 5.**
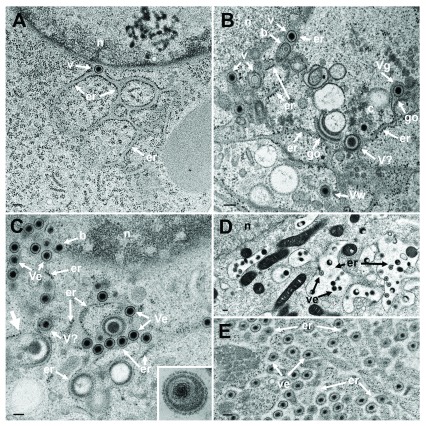
The effect of BFA. TEM of Vero cells at 9 hpi with R7041(ΔUs3) ( **A**), at 16 hpi ( **B**) and at 20 hpi ( **C**) with wt HSV-1, and at 15 or 17 hpi with wt HSV-1 and BFA exposure ( **D** and **E**). ( **A**) The ER (er) runs from the nucleus (n) towards the cell periphery forming an entity with the PNS that contains a virion (v). ( **B**) The ER contains virions. One capsid is in the stage of budding (b) into the ER. The ER continues into Golgi (go) membranes at two sites. One Golgi cisterna contains a virion (Vg), one virion has been derived by wrapping (Vw). Close to Golgi stacks, there is probably a virion (V?) of abnormal size. ( **C**) One capsid buds (b) at the nuclear (n) periphery. The ER is dilated and filled with virions (Ve) and dense material: An ER membrane turns into a Golgi membrane (thick arrow). ( **D**) After exposure to BFA from 8 to 15 hpi with wt HSV-1, the ER was dilated and contained some virions. ( **E**) The ER was almost filled with virions after exposure to BFA from 8 to 17 hpi with wt HSV-1. Note that virions in the PNS and ER are covered by a dense coat hiding spikes whereas spikes are clearly apparent on virions in the extracellular space ( **C** inset). Bars: 200 nm.

The Golgi complex undergoes dramatic changes during HSV-1 infection finally resulting in fragmentation and dispersion ( Campadelli *et al.*, 1993). However, the Golgi complex is not, or only minimal, involved in envelopment of R7041(ΔUs3) capsids ( Wild *et al.*, 2015). To identify ER-to-Golgi transitions, we thus imaged the Golgi complex in serial sections through wt HSV-1 or R7041(ΔUs3) or BoHV-1 infected cells by transmission electron microscopy after rapidly freezing and freeze-substitution applying a protocol especially suitable to visualize membranes ( Wild *et al.*, 2001). A series of images show that ER membranes continue into Golgi membranes. The ER runs from the perinuclear region into the membranes of the outermost Golgi stack ( Figure 4A). The very same membranes continue again into ER membranes so that this Golgi cisterna is interconnected between ER lamellae. The membranes of the adjacent stack also turn into ER membranes. ER membranes also pass somewhere into central regions of Golgi fields where they are devoid of ribosomes ( Figure 4BC). ER membranes may even connect two Golgi fields ( Figure 4D). The ER forms, as its name implies, a network ( Figure 4C) that connects to the PNS ( Figure 5A).

### Virions are within ER cisternae

**Figure 6.**
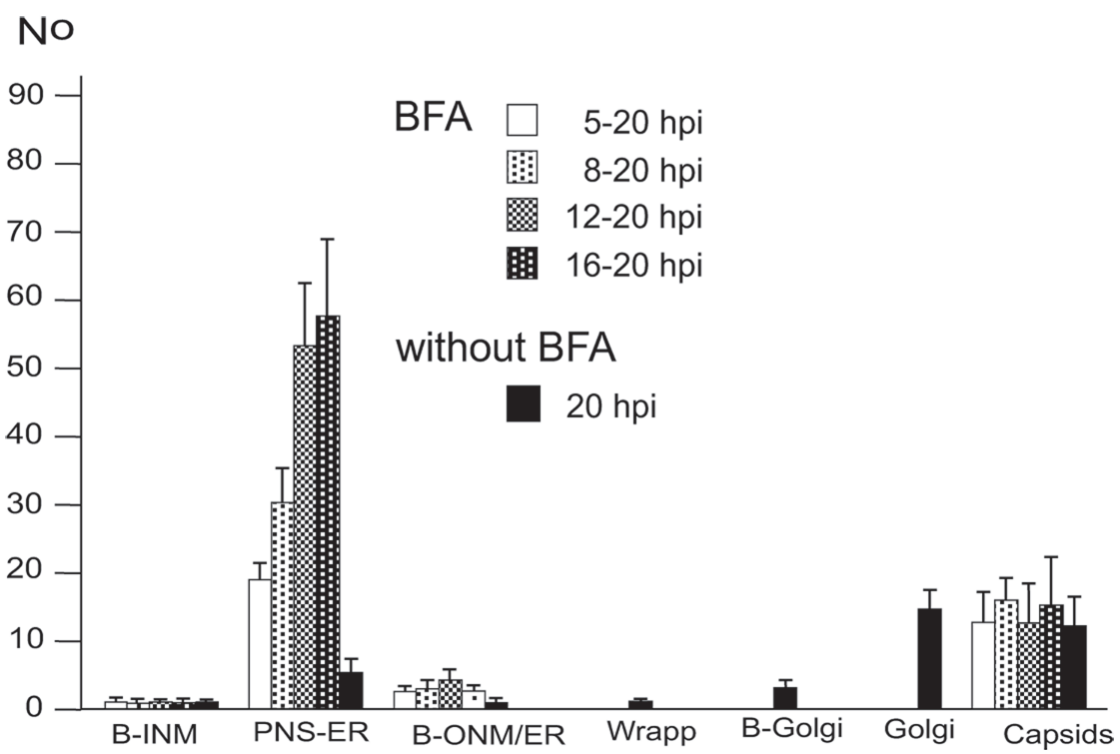
Means and standard deviations of the phenotype of HSV-1 infected Vero cells. BFA was added to monolayers at 5, 8, 12 or 16 hpi (MOI of 5) and incubated until 20 hpi. For control, inoculated cells were incubated for 20 h without addition of BFA. Cells were rapidly frozen at 20 hpi and processed for electron microscopy. The phenotypes of envelopment were counted in 10 cellular profiles of 5 independent experiments: Capsids budding at the INM (B-INM), at the ONM and ER membranes (B-ONM/ER) and at the Golgi complex (B-Golgi); virions in the PNS-ER compartment (PNS-ER); virions derived by wrapping (Wrapp); virions in Golgi cisternae or large vacuoles (Golgi); capsids in the cytoplasmic matrix (capsids).

Virions within the ER have been repeatedly shown, the first report dating back to the late 1960ties ( Schwartz & Roizman, 1969). Virions were in the PNS or anywhere in ER cisternae after HSV-1 infection ( Figure 5BC) as well as in Golgi cisternae of which membranes transit into ER membranes ( Figure 5B). Virions accumulate in the PNS-ER compartment late in infection ( Leuzinger *et al.*, 2005; Wild *et al.*, 2015), in the absence of Us3 ( Reynolds *et al.*, 2002; Wisner *et al.*, 2009) or gB/gH ( Farnsworth *et al.*, 2007) or after disintegration of the Golgi complex by BFA ( Chatterjee & Sarkar, 1992; Cheung *et al.*, 1991; Jensen & Norrild, 2002; Whealy *et al.*, 1991). To investigate the effect of BFA on virus release out of the PNS we exposed cells to BFA at 5, 8, 12 and 16 hpi with wt HSV-1, and harvested cells at 20 hpi for quantitative electron microscopic analysis and determination of infectious progeny virus. Electron microscopy revealed dilation of the PNS and ER containing many virions and amorphous material at 15 hpi ( Figure 5D). At 17 hpi, the ER was congested with virions ( Figure 5E). Quantitative analysis of phenotype distribution revealed that virions accumulate in the PNS-ER compartment after BFA administration in a time dependent manner. The number of intraluminal virions was 4 times higher when BFA was added at 8 hpi but 12 times higher than it was added at 16 hpi compared to that in untreated cells ( Figure 6). The number of virus particles interacting with the ONM and ER membranes in BFA exposed cells was about twice as high as in controls whereas the number of capsids in the cytoplasmic matrix did not differ significantly. We thus conclude i) that the interactions at the ONM and ER membranes are budding capsids contributing to accumulation of virions in the PNS and ER, ii) that more capsids bud at the ONM and ER membranes because no Golgi membranes are available after Golgi disintegration induced by BFA, iii) that inhibition of virion release out of the PNS-ER compartment is due to a blockage of the intraluminal transportation pathway after Golgi disintegration, and iv) that the Golgi complex delivered components to budding sites prior to its disintegration by BFA.

### Virions in the PNS-ER compartment are infective

**Figure 7.**
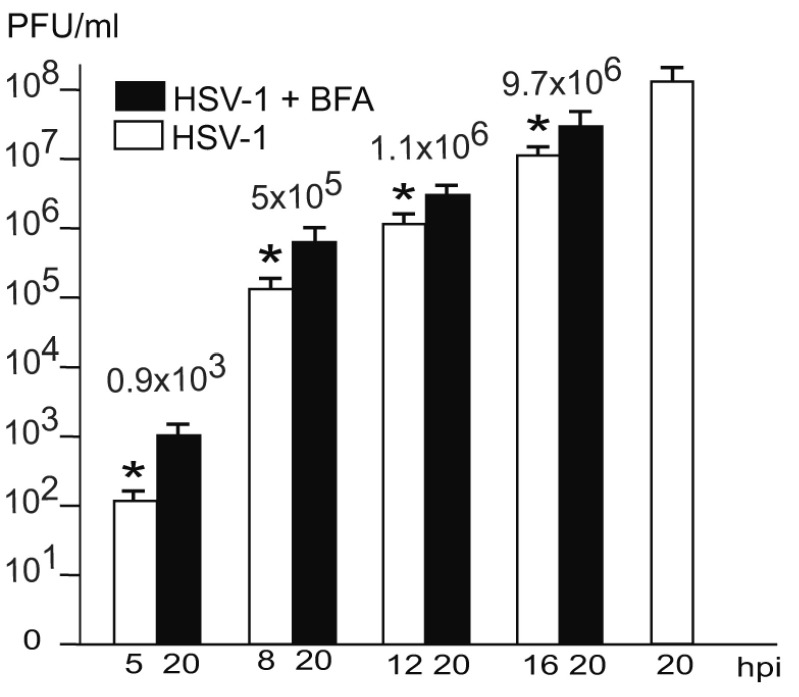
Virus yields at the time of BFA administration or controls (white) and at 20 hpi (black). The difference between virus yields (indicated with numbers) at the time of BFA addition and harvesting at 20 hpi is considered to be due to virus production after Golgi disintegration. These infectious virions correspond to the virions accumulating in the PNS-ER compartment. n = 4, p<0.01.

Us3 is not essential ( Poon *et al.*, 2006; Reynolds *et al.*, 2002; Ryckman & Roller, 2004; Wisner *et al.*, 2009). Us3 deletion mutants accumulating in the PNS are infective ( Wild *et al.*, 2015). Hence, it is reasonable to assume that wt HSV-1 virions in the PNS-ER compartment are also infective. To prove this idea, we determined infectious progeny virus by plaque titration at the time point of BFA administration and at 20 hpi. The Golgi complex completely disintegrates within less than 5 minutes after exposure of cells to BFA ( Hess *et al.*, 2000). Therefore, capsids cannot be enveloped by Golgi membranes anymore, or at least only for 5 minutes. As shown in Figure 6, there were no capsid interactions with cell membranes, such as Golgi membranes or endosomes whereas the PNS-ER compartment was full of virions. Nonetheless, infectious progeny virus was produced in a time depended manner. The later BFA was added the more infectious viruses were produced by 20 hpi ( Figure 7). Since virions accumulated invariably in the PNS-ER compartment in BFA exposed cells, and capsids were found to bud at the INM, ONM and ER membranes, we conclude that virions derived by budding at nuclear membranes and ER membranes are infective. The transport of capsids across the ONM generally believed to be fusion – de-envelopment – is budding as discussed below because of the phenotype and because it takes place also in the absence of the fusion proteins gB/gH.

### PNS, ER and Golgi complex form an entity

**Figure 8.**
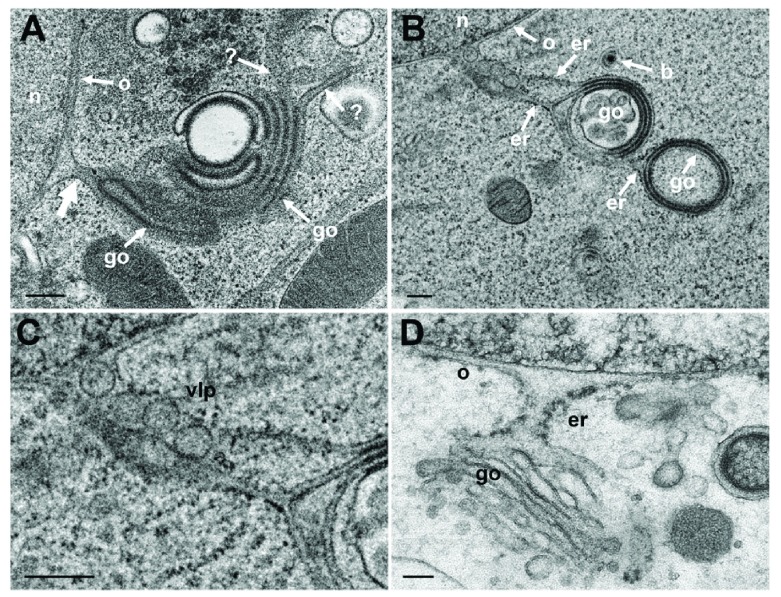
TEM of Vero cells at 12 hpi with R7041(ΔUs3) and of BoHV-1 infected MDBK cells, showing Golgi fields close to the nucleus (n). ( **A**) Golgi (go) membranes continue (thick arrow) into the ONM (o) as well as towards the cytoplasm indicated by (?) because the destination is unknown. ( **B**) Golgi membranes continue via ER membranes (er) into the ONM. The ER contains 4 virus-like particles. ( **C**) Details of panel **B**. ( **D**) PNS, ER and Golgi complex form an entity in a BoHV-1 infected MDBK cell ( **D**: This figure has been reproduced with permission of P. Wild *et al.*, Micron 33, 2002, Elsevier). Bars 200 nm.

The nuclear envelope is part of the ER. The outer nuclear membrane (ONM) is stubbed with ribosomes. The ONM continues into ER cisternae, which, in turn, merge with Golgi cisternae. Golgi cisternae were also found to connect to the PNS via short ER-Golgi intermediates in R7041(ΔUs3) infected Vero cells ( Figure 8ABC) and BoHV-1 infected MDBK cells ( Figure 8D). ER-Golgi intermediates contained virus like particles ( Figure 8B). The continuum between PNS and Golgi cisternae is considered likely to serve as a direct, short and efficient pathway to transport virions form the site of budding to Golgi cisternae for packaging.

### Virions are within Golgi cisternae and/or vacuoles

**Figure 9.**
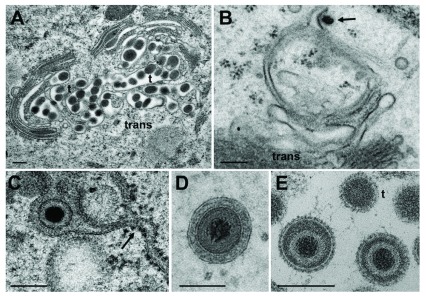
Virions in Golgi cisternae versus budding of capsids. ( **A**) Golgi cisternae engulfing BoHV-1 virions at 20 hpi. Many of them are tangentially (t) sectioned. ( **B**) Budding BoHV-1 capsid at a Golgi membrane of the cis-face (arrow). ( **C**) HSV-1 virion in a Golgi cisterna that connects to the ER (arrow). Note the dense content within the ER and Golgi cisterna indicating little loss of material during processing. ( **D**) Concentric vacuole derived by wrapping containing a single BoHV-1 virion. The space between viral envelope and vacuolar membrane is always filled in well preserved cells. ( **E**) Virions in a large vacuole or cisterna exhibiting clearly spikes even in tangentially (t) sectioned virions. Bars: 200 nm.

Capsids are postulated to be enveloped at the *trans* Golgi network ( Mettenleiter *et al.*, 2006) by a process designated wrapping. However, capsids can bud at any location of the Golgi complex ( Figure 9B) and vacuoles as have been shown for HSV-1 ( Leuzinger *et al.*, 2005; Stannard *et al.*, 1996), BoHV-1 ( Wild *et al.*, 2002) and pseudorabies virus ( Klupp *et al.*, 2008), and even at endosomes ( Albecka *et al.*, 2016; Hollinshead *et al.*, 2012). The result of wrapping is a small concentric vacuole ( Figure 9D) containing a single virion as shown elsewhere in detail ( Leuzinger *et al.*, 2005; Wild *et al.*, 2005). Golgi cisternae and vacuoles can contain one to numerous virions in a given section plane ( Figure 9A). The cavities at the *trans*-face of the Golgi complex in Figure 9A are more likely to represent cisternae rather than vacuoles because of their shape and location. The virions had gained access either by budding or by intraluminal transportation via ER-Golgi intermediates, as might be the case also in Figure 9C. Note that the viral envelope including spikes are covered by a dense layer in narrow Golgi cisternae ( Figure 9C) whereas spikes are visible on virions in wide Golgi cisternae or large vacuoles ( Figure 5 inset) and in the extracellular space ( Figure 9E) as shown previously ( Leuzinger *et al.*, 2005; Wild *et al.*, 2005). From the facts that virions are within ER and Golgi cisternae, and that the ONM continues via ER membranes into Golgi membranes forming an entity, we postulate that virions can be intraluminally transported from the PNS via ER into Golgi cisternae.

### ER-to-Golgi transitions in uninfected cells

**Figure 10.**
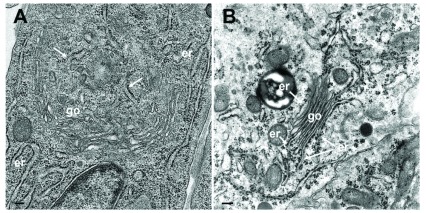
( **A**) TEM image of a cultured epithelial cell in which the Golgi complex is embedded in the ER. The ER membranes (arrows) run into the Golgi complex, close to a structure that probably represents a tangential section of the Golgi organizing center. ( **B**) TEM image of a parathyroid cell prepared according to conventional protocols showing Golgi membranes (go) continuing into ER membranes (er). Bars: 200 nm.

ER-to-Golgi transitions are not only established in infected cells, but also in cultured epithelial cells ( Figure 10A) or in cells in organs, e.g. parathyroid gland, which was prepared by perfusion fixation ( Wild *et al.*, 1985) according to conventional protocols ( Figure 10B). These observations suggest that ER to Golgi transitions may also serve as a direct transportation route e.g. for proteins to be finally released by exocytosis.

Raw images for Figure 1–Figure 5, Figure 8–Figure 10 Click here for additional data file. Copyright: © 2018 Wild P et al. 2018 Data associated with the article are available under the terms of the Creative Commons Zero "No rights reserved" data waiver (CC0 1.0 Public domain dedication).

Raw values for Figure 6 and Figure 7 Click here for additional data file. Copyright: © 2018 Wild P et al. 2018 Data associated with the article are available under the terms of the Creative Commons Zero "No rights reserved" data waiver (CC0 1.0 Public domain dedication).

## Discussion

**Figure 11.**
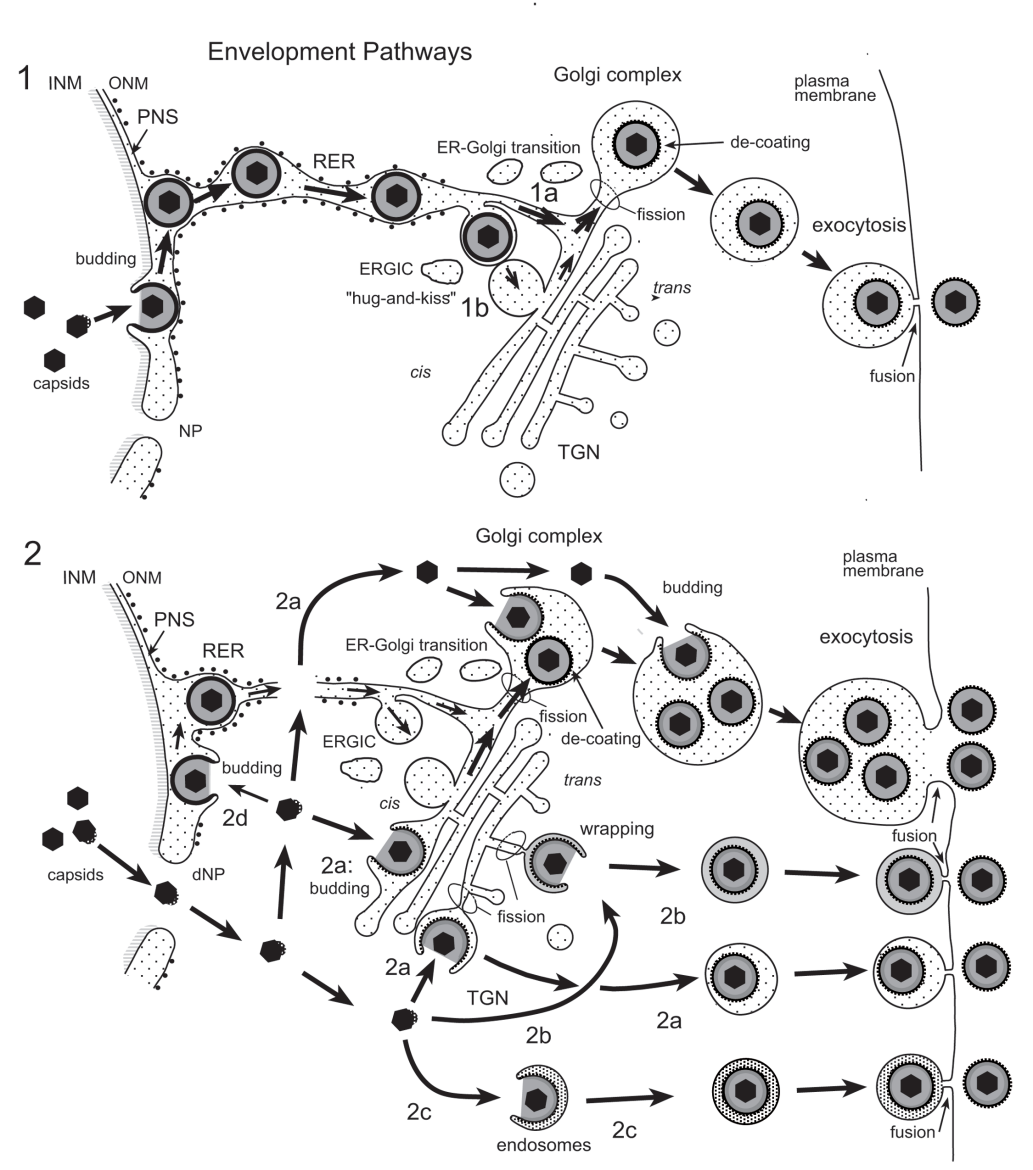
Schematic representation of proposed herpes virus envelopment pathways. (1) Capsids bud at the INM into the PNS acquiring tegument and an envelope covered with a dense coat. These perinuclear virions are transported into the RER and further via Golgi transitions (1a) or the ERGIC (“hug-and-kiss”, 1b) into Golgi cisternae where they are packaged into transport vacuoles, which are detached from Golgi membranes by fission. The dense coat is shed off while vacuoles are transported to the cell periphery for exocytotic release of uncoated virions into the extracellular space. (2) Capsids gain direct access to the cytoplasmic matrix via dilated nuclear pores (dNP), and are transported to any site of the Golgi complex. They either bud into Golgi cisternae and vacuoles, respectively (2a) or are enveloped by a process designated wrapping (2b) that involves budding and concomitant formation of a small transport vacuole engulfing a single virion. Capsids can also be enveloped by endosomal membranes (2c). Occasionally, capsids may bud at the OM or RER (2d), and the resulting virions are intraluminally transported as in pathway 1. Finally, vacuoles derived by fission from Golgi membranes or from membranes of vacuoles or endosomes transport virions to the cell periphery and release them into the extracellular space via exocytosis. The dense coat, which derived during the budding process at the INM and ONM and probably protects the viral envelope from fusion with membranes the virions are transported along, is shed of (de-coating) in transport vacuoles at latest when virions are released into the extracellular space. During budding at Golgi cisternae and vacuoles, a dense rim of tegument is closely attached to the inner layer of the viral envelope. However, no dense coat is formed so that spikes (glycoproteins) are readily seen in high resolution micrographs.

The Golgi complex is among the first organelles that rapidly disintegrate during processing for electron microscopy after improper fixation and processing ( Han *et al.*, 2013; Wild *et al.*, 2001). To minimize disintegration, we employed a technique that leads to improved retention of cellular material ( Cope & Williams, 1969; Weibull *et al.*, 1984), and to improved spatial and temporal resolution ( Mueller, 1992). This is especially important for analytical studies of cells in which the Golgi complex is involved in highly dynamic processes such as packaging of proteins into granules ( Ellinger *et al.*, 2010; Orci *et al.*, 1981; Wild *et al.*, 1982) in the secretory pathway, or envelopment of capsids and vacuole formation ( Figure 11) for delivery of hundreds of virions to the cell periphery ( Leuzinger *et al.*, 2005; Wild *et al.*, 2015; Wild *et al.*, 2002). Because of the difficulties in preservation of the Golgi ultrastructure ( Ellinger *et al.*, 2010; Han *et al.*, 2013; Wild *et al.*, 2001), and because of its complexity, three-dimensional structure is poorly understood. Cryo-FESEM revealed the Golgi complex to be a complex tightly packed structure. The membrane of the outermost cisternae may completely cover the *cis*-face. TEM also revealed that the Golgi complex is embedded in the ER system, with multiple membrane connections forming a Golgi-ER entity.

The Golgi complex fragments and disperses about 16 hpi with HSV-1 ( Campadelli *et al.*, 1993) adding additional difficulties for understanding Golgi function in herpes virus envelopment. Simultaneous immune labelling revealed that the *cis*-Golgi had disappeared by 16 hpi with wt HSV-1 whereas the *trans*-Golgi had fragmented. In contrast, the *cis*- and *trans*- Golgi had enlarged already by 6 hpi with the Us3 deletion mutant R7041(ΔUs3), which is related to the enhanced phospholipid synthesis in the absence of Us3 ( Wild *et al.,* 2012). The significance of Us3 on the Golgi complex is under study. To address the significance of the Golgi complex in virus envelopment and virus transportation, we thus investigated infected cells between 8 hpi (the approximate time of onset of envelopment) and 16 hpi. Our data clearly show that the Golgi complex is localized in a juxtanuclear position appearing as a compact entity by cryo-FESEM. Golgi membranes continue into ER membranes which in turn connect to the ONM forming a continuum between Golgi cisternae and PNS. Thus, the presence of virions within the PNS, ER cisternae and Golgi cisternae strongly suggests that the ER-to-Golgi transition is used as a direct intraluminal pathway to deliver virions from the PNS into Golgi cisternae ( Figure 11, pathway 1). This idea is supported by the fact that about 80 HSV-1 virions per mean cell volume were within ER cisternae at 12 and 16 hpi but close to 300 by 24 hpi ( Wild *et al.*, 2015) suggesting that virus transportation out of the ER is inhibited after Golgi fragmentation ( Campadelli *et al.*, 1993). Virus transportation out of the ER is also drastically inhibited after BFA exposure. The ER dilates and secretory protein transport from the ER to the Golgi complex is impeded after BFA treatment ( Fujiwara *et al.*, 1988; Misumi *et al.*, 1986). Hence, the integrity of the Golgi complex is crucial for export of both secretory proteins and HSV-1 out of the ER suggesting that HSV-1 release from the ER to the Golgi complex follows a similar pathway as secretory proteins either by vesicle formation involving cop II ( Klumperman, 2000) or equivalent, or via ER-Golgi transitions.

According to the currently stressed herpes virus egress theory ( Mettenleiter *et al.*, 2013), formation of infectious herpes viruses follows a complicated uneconomic pathway involving primary envelopment by budding of capsids at the INM, de-envelopment of capsids by fusion of the viral envelope with the ONM releasing capsids and tegument into the cytoplasmic matrix, and re-envelopment by wrapping at the *trans* Golgi network. The interaction of the viral envelope with the ONM was described the first time in 1968 ( Darlington & Moss, 1968) and identified as budding of capsids from the cytoplasmic matrix into the PNS. About 30 years later, it was tried to prove that this process is fusion ( Kopp *et al.*, 2002; Naldinho-Souto *et al.*, 2006; Skepper *et al.*, 2001). Fact is that the phenotypes of the process taking place at the ONM are identical with those at the INM ( Darlington & Moss, 1968; Leuzinger *et al.*, 2005; Wild *et al.*, 2005; Wild *et al.*, 2012) and that they show all characteristics of budding. Budding requires proteins that are able to induce positive and negative curvatures. Budding at the INM is driven by UL31/UL34 ( Bigalke & Heldwein, 2016; Bigalke & Heldwein, 2017; Bigalke *et al.*, 2014; Hagen *et al.*, 2015). UL31 and UL34 are also present at the ONM even in cells infected with Us3 deletion mutants ( Reynolds *et al.*, 2002) those envelopes are unable to fuse with the ONM ( Reynolds *et al.*, 2002; Wisner *et al.*, 2009). Therefore, the presence of UL31/UL34 at the ONM cannot be the result of membrane transportation from the INM to the ONM via budding and subsequent fusion as often used as arguments for the presence of viral proteins at the ONM ( Skepper *et al.,* 2001). Furthermore, UL34 was shown to localize at the ER ( Yamauchi *et al.*, 2001) and that UL31 is required for its dislocation to the INM.

Us3 deletion mutants arising by budding at the INM cannot be de-enveloped at the ONM and, hence, accumulate in the PNS. Nonetheless, Us3 deletion mutants are infective ( Reynolds *et al.*, 2002; Ryckman & Roller, 2004; Wild *et al.*, 2015) implying that all essential proteins must have been transported to the nuclear periphery so that they can become part of virions deriving by budding at the INM. The same is considered likely to be true for wt HSV-1 virions because they differ from Us3 deletion mutants only in the ability to be released out of the PNS. Indeed, by constructing a triple-fluorescent recombinant it was shown that the capsids fusion protein VP26 localized in the nucleus, the tegument fusion protein VP16 and the envelope glycoprotein H localized at the nuclear rim early in infection ( de Oliveira *et al.,* 2008). In VZV, it was shown that biosynthesis of glycoprotein starts within 2 h after infection ( Yao *et al.,* 1993). Production of infectious progeny virus after exposure cells to BFA indicate that they have derived by budding at nuclear membranes and ER membranes see ( Figure 6), and that e.g. glycosylation of glycoproteins had to take place prior to disintegration of the Golgi complex. The glycoprotein gH was detected in the cytoplasm as well as at the nuclear periphery at 4 hpi, ( de Oliveira *et al.,* 2008), i.e. much earlier prior to the onset of budding. Therefore, we assume that infectious virions can be produced after administration of BFA at 5 hpi by budding at nuclear membranes though at a limited number. The later cells were exposed to BFA the more infectious virions were produced suggesting that the later the Golgi complex disintegrated the more proteins and lipids could have been produced. Considering fragmentation of the Golgi complex by about 16 hpi with wt HSV-1 ( Campadelli *et al.,* 1993) the question arises where are the large number of virions produced after Golgi fragmentation?

**Figure 12.**
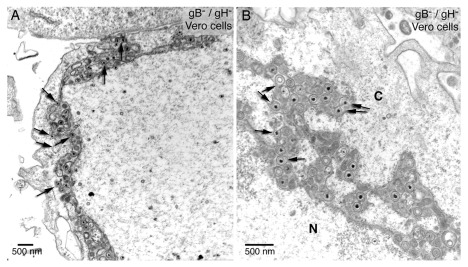
Virus transport across the ONM in the absence of the fusion proteins gB/gH. gB/gH null virions accumulate in the PNS-ER compartment. The arrows (inserted by P. Wild) point to interactions of virus particles with the ONM and ER membranes showing the characteristics of budding. The fusion proteins gB/gH are missing. Therefore, these virus membrane interactions represent various stages of budding – not of fusion. Reproduced from Farnsworth *et al.*, 2007, Figure 2), copyright (2007) National Academy of Sciences, U.S.A.

 For de-envelopment via fusion of the viral envelope with the ONM fusion proteins would be required. Theglycoproteins B and H (gB/gH), members of the quartet of fusion proteins responsible for cell entry ( Turner *et al.*, 1998), have been claimed to be responsible for fusion of the viral envelope with the ONM ( Farnsworth *et al.*, 2007). Indeed, they clearly showed by electron microscopy striking interactions with the ONM and ER membranes in cells infected with a gB/gH deletion mutant. The crux is, first, that the viral envelope cannot fuse with ONM in the absence of gB/gH, second, numerous virions accumulated in the PNS and adjacent ER cisternae and, third, none the virus membrane interactions exhibit characteristics of fusion. Therefore, the phenotypes shown in Figure 12, a reproduction of Figure 2 of this report ( Farnsworth *et al.*, 2007) with permission of the copyright holder, represent undoubtedly various stages of capsids budding from the cytoplasmic matrix into the PNS and ER cisternae. Furthermore, in Vero cells infected with the gB/gH deletion mutant, three times more virions were found in the cytoplasm compared to wild type infected Vero cells, and about a third in the extracellular space. Similar relations though less pronounced were found in other cell types. Taken together, gB/gH are not involved in release of virions out of the PNS via fusion. The process taking place at the ONM claimed to be fusion is budding absolutely implying another transportation route than that of de-envelopment by fusion of the viral envelope with the ONM.

Wrapping demands an enormous amount of membranes that would need to be provided to the TGN, which, to our knowledge, is not clear yet. However, capsids can be wrapped at diverse sides of the Golgi complex or can bud into Golgi cisternae and/or vacuoles via another pathway than wrapping. There are numerous reports showing HSV-1 virions in vacuoles and/or Golgi cisternae ( Homman-Loudiyi *et al.*, 2003; Leuzinger *et al.*, 2005; Stannard *et al.*, 1996; Sutter *et al.*, 2012; Wild *et al.*, 2015; Wild *et al.*, 2002). Budding of capsids at various sites of Golgi membranes as well as virions in Golgi cisternae and vacuoles were also shown in pseudorabies virus infected cells ( Klupp *et al.*, 2008). These virions in all these vacuoles and/or Golgi cisternae did not arise by wrapping because wrapping results in a single virion in a concentric vacuole as shown in Figure 9D and described in detail ( Wild *et al.,* 2005). They may have entered Golgi cisternae by budding into them or by intraluminal transportation via ER-to-Golgi transitions. Transient elements from *cis* and *trans* Golgi sides have been shown in various cells ( Pavelka & Roth, 2015). It was also suggested that the *cis*-Golgi approaches the ER and contacts the ER exit sites in the yeast Saccharomyces cerevisiae to capture cargo for transportation to the Golgi complex ( Kurokawa *et al.*, 2014). This ‘hug-and-kiss’ behavior could be another route to transfer virions, which are intraluminally transported to ER exit sites, into the Golgi cisternae.

In cells prepared for improved resolution, we found no valid arguments for de-envelopment by fusion of the viral envelope with the ONM. There are a number of facts that argue clearly against the de-envelopment theory. First, the morphology of the process taking place at the INM and ONM are identical showing all characteristics for budding ( Darlington & Moss, 1968; Leuzinger *et al.*, 2005; Wild *et al.*, 2005; Wild *et al.*, 2012) but none for fusion ( Haluska *et al.*, 2006; Kanaseki *et al.*, 1997) considering fundamentals of membrane bound transportation ( Leabu, 2006; Peters *et al.*, 2004; White, 1992). Second, virions have been repeatedly shown within ER cisternae ( Gilbert *et al.*, 1994; Granzow *et al.*, 1997; Leuzinger *et al.*, 2005; Radsak *et al.*, 1996; Schwartz & Roizman, 1969; Stannard *et al.*, 1996; Sutter *et al.*, 2012; Wild *et al.*, 2002). To reach this location, either virions need to be transported from the PNS into the ER, or capsids have to bud from the cytoplasmic matrix into the ER. If virions can be intraluminally transported out of the PNS the viral envelope must be protected from fusion with membranes the virions are transported along. If capsids have the ability to bud at ER membranes capsids are very likely to be able to bud at the ONM since the ONM is part of the ER. Third, virions can accumulate to large numbers in the PNS, e.g. in the absence of the protein kinase Us3 ( Poon *et al.*, 2006; Reynolds *et al.*, 2002; Wild *et al.*, 2015). Strangely enough, these virions are infective ( Reynolds *et al.*, 2002; Ryckman & Roller, 2004; Wild *et al.*, 2015) despite the inability of the envelope to fuse with the ONM ( Wisner *et al.*, 2009) clearly contradicting the theory that de- and re-envelopment is essential to become infective ( Klupp *et al.*, 2011). Fourth, the equivalent to budding is the formation of coated pits resulting in coated vesicles ( Owen & Luzio, 2000; Pearse *et al.*, 2000). Coated vesicles derive e.g. from the plasma membrane and are transported towards the Golgi complex where they fuse with Golgi membranes ( Orci *et al.*, 1981). However, coated vesicles must be uncoated to gain the ability for fusion. The coat consists of clathrin that drives formation of coated pits and finally coated vesicles and protects them from fusion. Clathrin was claimed to be involved in envelopment of HSV-6 capsids at Golgi membranes together with viral proteins ( Mori *et al.*, 2008). Budding of HSV-1 capsids at the INM is driven by the nuclear envelopment complex, UL31/UL34, ( Bigalke & Heldwein, 2015; Bigalke *et al.*, 2014; Hagen *et al.*, 2015) located at the nuclear rim ( Mou *et al.*, 2009).

Budding at the INM, ONM and ER membranes starts with deposition of dense substances that finally result in a dense coat at the viral envelop. The dense coat is readily seen on virions in the PNS, ER and, inconsistently, in Golgi cisternae and large vacuoles containing many virions. The dense coat suggests that it protects virions from fusion with membranes the virions is transported along but allows virus transportation from the PNS into ER and Golgi cisternae. However, in large Golgi cisternae and/or vacuoles, many virions are without dense coat. Instead, spikes are visible like at virions in the extracellular space. Budding at Golgi membranes takes place without dense coat formation.

## Conclusions

Golgi membranes interconnect with ER membranes in cells infected with HSV-1, a Us3 deletion mutant thereof or with BoHV-1 as well as in uninfected cells. The ER continues into the ONM, that turns into the INM at sites of nuclear pores. Consequently, the PNS, ER and Golgi complex forms an entity that can be only visualized by TEM, either when the membranes of all three compartments are luckily hit in the same plane of a given section, or by 3D-reconstruction after imaging of serial sections, focused ion beam (FIB) microscopy or electron tomography. Fact is that virions are intraluminally transported out of the PNS into the ER. This implies that the viral envelope needs to be protected from fusion with the membranes the virions are transported along. Thus, the significance of the dense coat, which derives during budding of capsids at nuclear membranes, is protecting the viral envelope from fusion in a similar manner as clathrin protects coated vesicles from fusion. The ER-to-Golgi transitions, virions in Golgi cisternae with a similar dense coat as virions in the PNS and ER, and inhibition of virion transportation out of the PNS and ER after disruption of the Golgi complex by BFA, strongly suggest that virions are intraluminally transported from the PNS through the ER into Golgi cisternae. The process at the ONM reported to be fusion is budding that takes place in the absence of the fusion proteins gB/gH. Therefore, we propose, first, that intraluminal transport is the only pathway for virions out of the PNS, and, second, that capsids gain direct access to the cytoplasm from the nucleus via dilated nuclear pores and impaired nuclear envelope, and are than enveloped by membranes of the Golgi complex, of vacuoles or endosomes The function of gB/gH and other proteins, such as the Us3 kinase, need to be reinvestigated and discussed in an unbiased context of herpes virus morphogenesis and egress.

## Data availability

The data referenced by this article are under copyright with the following copyright statement: Copyright: © 2018 Wild P et al.

Data associated with the article are available under the terms of the Creative Commons Zero "No rights reserved" data waiver (CC0 1.0 Public domain dedication). 

Dataset 1: Raw images for Figure 1– Figure 5, Figure 8– Figure 10. DOI, 10.5256/f1000research.12252.d179644 ( Wild *et al.*, 2017a)

Dataset 2: Raw values for Figure 6 and Figure 7. DOI, 10.5256/f1000research.12252.d179645 ( Wild *et al.*, 2017b)
