# Supplementary material for: Endoplasmic reticulum-to-Golgi transitions upon herpes virus infection
Source: F1000Res. 2018 Feb 28;6:1804. Originally published 2017 Oct 5. [Version 2] doi: 10.12688/f1000research.12252.2 (PMC6080407; doi:10.12688/f1000research.12252.2)
Supplement: Peer review history

# Peer review: Referee response for version 2

**DOI:** 10.5256/f1000research.15292.r32782

**Published:** 2018-04-13

**Authors:** José A Martínez-Menárguez

**Affiliations:** 1 Department of Cell Biology and Histology, Biomedical Research Institute of Murcia, University of Murcia, Murcia, Spain

The manuscript by Wild and colleagues analyzes the mode of transport of herpes virus by using morphological techniques. The main result is that virions are intraluminally transported from the PNS –ER to Golgi cisterna via direct continuities. The topic is very interesting and the authors show nice images. However, in my opinion, the results are over interpreted. Many of the conclusions cannot be obtained without more specific techniques such as immunoelectron microscopy and electron tomography. Thus, the interest of the paper is limited.

 Major criticisms and some minor points:

 First, the manuscript is difficult to read and hard to understand for not specialists, although Figure 11 helps a lot. It is also difficult to understand the aim of the work in the Introduction section and what it is really new. Thus, I strongly recommend to authors to clarify these points and simplify the text.

 Title and text. It is confusing the term “endoplasmic reticulum-to-Golgi transitions”.  The term transition is ambiguous and could also be used for vesicular transport. In order to clarify it is better to use the terms connections or direct continuities.

 The abbreviation PNS should be specified the first time in text (introduction section, second paragraph)

 Results. 1 ^st ^paragraph. The title of the paragraph is not informative. It is well known that “the Golgi complex is a tightly packed entity situated close to the nucleus”

 Results and Figure 1. Note that TGN46 is a marker of the TGN46 rather than trans-Golgi (Prescott et  al Eur J Cell Biol. 1997 72:238-46). This point may be important for interpreting the confocal experiments. I don’t see big differences between the size of the Golgi complex in Figures 1B and 1C. Perhaps, it should interesting to show more cells (or quantitative analysis).

 Results and Figures 2 and 3. It could be interesting to show the Golgi complexes of control cells in order to compare how infection affects its morphology. Moreover, the use of immunocytochemycal techniques with Golgi markers will help to interpret your results. For instance, this methodology can confirm that the outer-most cisterna is, in fact, the cis-most cisterna. It is very surprising that this membrane is unusually “smooth” with very few fenestrations. Did you see in these 3D images connections of this membrane with the ER? Is it possible that these Golgi are autophagosomes?

 Results and Figure 4. How do you known that the membrane showed in Figure 4A (and 5B) is a Golgi cisterna? It is hard to believe without immnuoelectron microscopy. In fact, it could be just a ribosome-free region of ER. It is hard to believe from these pictures that “ER membranes continue into Golgi membranes”. The relationship between ER and Golgi membranes showed in Figures 4C and 4D is not different than the describe for most cell types, even the stack looks very “normal”.

 Results and Figure 5D. Are you sure that these membranes are ER? So that, why are they so different than those showed in figure 5E? Is it possible that these structures are the same than the vacuoles showed in Figure 9A?

 Figures 8A-C. These figures are very interesting but again, without immunocytochemistry and 3D analysis is difficult to interpret. It should be noted that, in addition to direct continuities, the existence of indirect transport by large transport intermediates cannot be excluded. Figure 8D (and 12) is very nice but “is already published”. For me (I don’t know for the journal) it does not have any sense to include published images

 Text and Figure 9A. Why do you think that these structures are cisterna rather than vacuoles? For me, it is evident that these membranes are not cisterna. In fact Golgi cisternae look quite normal although they show a few distensions. Again, are you sure than Figure 9C is a Golgi cisterna?

 Figure 10 and the last paragraph of the result section. These figures are absolutely unnecessary. The relationship between the ER and the cis and trans cisternae it is well known many decades ago.

I have read this submission. I believe that I have an appropriate level of expertise to confirm that it is of an acceptable scientific standard, however I have significant reservations, as outlined above.

# Peer review: Referee response for version 2

**DOI:** 10.5256/f1000research.15292.r32673

**Published:** 2018-04-10

**Authors:** Alexandre A. Mironov

**Affiliations:** 1 FIRC Institute of Molecular Oncology Foundation (IFOM), Milan, Italy

The paper by Wild et al studies the question of how Herpes virus is assembled and transported. The paper is important but not conclusive. I agree with the authors that in the literature, there are no convincing images demonstrating the nucleocapsis being attached to the cytosolic part of the external membrane of the nuclear envelope. Such image would suggest that virion fused with the external membrane of the nuclear envelope and then would be released to the cytosol. If such mechanism exists, there should be many such situations and many nucleocapsids in the cytosol. Moreover, there should be a mechanism involved into the direct delivery of nucleocapsid towards the Golgi.

 However, there is only one image (Fig. 9A) demonstrating (although not convincingly) budding BoHV-1 capsid into the lumen of the Golgi cisterna. Appearance of the capsid in the cytosol could be a result of enlargement of nuclear pores. It is not clear whether virions are transported through the Golgi. If such, there should be indications that proteins of virions undergo glycosylation with Golgi enzymes. If these proteins have specific type of glycosylation similar to that in CFTR there could be transport of virion through the Golgi without integration of virion containing vacuoles into Golgi cisternae.

 In general, there could be the following possibilities for the acquisition of the limiting membranes by virions.

 1. Negative budding into the lumen of the nuclear envelope through the inner membrane, then fission and formation of almost-ready virion inside the lumen of the nuclear envelope, subsequent ER-Golgi transport, glycosylation within the Golgi complex, transport from the Golgi complex towards the PM in Golgi-to-PM carriers, fusion of the most external membrane of carrier and release of the virion into the extracellular space. If virions already surrounded by limiting membranes were transported from the nuclear envelope towards the Golgi complex, there should be mechanisms for such transfer. For instance, within the cytosol there are microtubules and MT-based motors, or actin-dependent comets and so on.

 2. Negative budding into the lumen of the nuclear envelope through the inner membrane, then fission and formation of almost-ready virion inside the lumen of the nuclear envelope; fusion of the limiting membrane of virion with the external membrane of the nuclear envelope and release of the capsid without virion matrix into the cytosol and then budding into the Golgi cisterna distension and the subsequent intra-Golgi transport, Golgi glycosylation and post-Golgi transport as in the first pathway. In this case there should be a mechanism for the fusion of virion with the external membrane of the nuclear envelope. Also one should present several images showing the situations when the external membrane of the nuclear envelope contain invaginations (buds) inside the lumen of the nuclear envelope and the capsid is open towards the cytosol and also images where a capsid forms an intraluminal bud inside the distension of the Golgi cisternae.

 3. Negative budding into the lumen of the nuclear envelope through the inner membrane, then fission and formation of almost-ready virion inside the lumen of the nuclear envelope; fusion of the limiting membrane of virion with the external membrane of the nuclear envelope and release of the capsid without virion matrix into the cytosol and then transport from the nucleus toward the PM and formation of bud on the PM with subsequent blebbing (external budding). One should present several images showing the situations when the external membrane of the nuclear envelope contain an invagination (bud) into the lumen of the nuclear envelope and the capsid is open towards the cytosol and images with the capsid inside the blebs of the PM.

 4. Movement of capsids through widened nuclear pores into the cytosol, transport from the nucleus toward the PM and formation of bud on the PM with subsequent blebbing (external budding). One should present images with the capsid inside the blebs of the PM and a capsid within enlarged nuclear pore or extremely close to the widened pore.

 5. Movement of capsids through widened nuclear pores into the cytosol, transport from the nucleus toward the Golgi complex and then budding into the Golgi cisterna distension and subsequent intra-Golgi and post-Golgi transport as in the first pathway. One should present images with a capsid within enlarged nuclear pore or extremely close to the widened pore and images with a capsid, which forms an intraluminal bud inside the distension of the Golgi cisternae.

 Thus, it is necessary to study the type of glycosylation of virion trans membrane proteins, which are inserted into the external virion membrane. It is necessary and it could be nice to perform 3DEM in order to solve all these unclear questions. Also in order to solve questions mentioned in this paper, it is necessary to test the role of membrane fusion (microinjection of aSNAPmutant or treatment of cells with BAPTA-AM) for the intracellular transport of virions. ER-Golgi and intra-Golgi transport of such large virions should be dependent on membrane fusion because there are no convincing data showing that connections between Golgi cisternae are stable (Mironov et al., 2016; 10.1016/j.tice.2016.12.002). The most probable mechanism is the kiss-and-run.

 Minor points

 1. Images in Fig. 2, 5, 8, 9 are too small for analysis by eyes. Only at 5-fold enlargement of the figure on computer some details become visible. It is not clear why the journal does not use its server for the images with normal size. It is almost impossible to see detail in Fig. 12.

 2. It is not completely clear the role of experiments with brefeldin A and with Us3 deletion mutant.

 3. I agree with Dr. Griffiths that the Golgi complex is well preserved with chemical fixation high pressure freezing without three-dimensional may be not so important.

 4. In page 5, the authors write: "A series of images show that ER membranes continue into Golgi membrane". It is necessary to make 3D EM (Mironov et al., 2016 Tissue and Cell. 10.1016/j.tice.2016.12.002) to prove this. In fact, the authors demonstrated continuation only between the ER and trans-ER cisternae.

 5. In Fig. 11/2 the most trans cisterna (TMC) is drawn incorrectly (see Ladinsky et al., 1994 J Cell Biol. 127(1):29-38). There are no protrusions from the plane part of TMC. These protrusions exist but only from the rims.

 6. After careful examination of images of the paper at 4-fold magnification I found that in Fig. 4A, instead of the Golgi complex the early autophagosome is shown.

 7. In Fig. 4B, there are only two GCs but not four. The Golgi complexes are visible in the in the central upper zone centre upper and the in the left lower zone. In the upper left and upper right zones, MVBs are shown.

 8. Figure 8D in Wild et al. (2018) is the exact copy of Figure 2F from Wild et al. (2002).

I have read this submission. I believe that I have an appropriate level of expertise to confirm that it is of an acceptable scientific standard, however I have significant reservations, as outlined above.

# Peer review: Referee response for version 2

**DOI:** 10.5256/f1000research.15292.r31302

**Published:** 2018-03-16

**Authors:** Gareth Griffiths

**Affiliations:** 1 Department of Biosciences, University of Oslo, Oslo, Norway

I have read this submission. I believe that I have an appropriate level of expertise to confirm that it is of an acceptable scientific standard.

# Peer review: Referee response for version 1

**DOI:** 10.5256/f1000research.13264.r26714

**Published:** 2017-10-20

**Authors:** Gareth Griffiths

**Affiliations:** 1 Department of Biosciences, University of Oslo, Oslo, Norway

The paper by Wild and colleagues investigates the question of how Herpes virus assembles, a story that has been under debate for decades. This family of DNA viruses assemble in the nucleus and thereafter opinions differ as to how the virus gets out of the cell. The bulk of the data are based on high pressure freezing and freeze substitution and are of exceptionally high quality.

 However, I had a hard time trying to figure out what the point of the paper was. The title suggests a virus-induced ‘transition’ between ER and Golgi. I don't understand what they conclude about the ER-Golgi nor what it means for the virus. Virus-induced implies a difference between infected and uninfected cells. The only image of an uninfected cell is the IF image in Fig 1. I don't see much difference in the pattern of GM-130 labeling, a marker of early Golgi compartments. They show no EM images of uninfected cell Golgi.

 I had a serious problem also trying to understand how they could distinguish between the three models shown in Fig 11. I could not follow their logic. In my opinion to do this in a satisfactory was they would need to use host cell markers for the different Golgi compartments with use of some viral membrane markers. To be able to give directionality to their static images they would also need e.g an inducible system whereby they could block some stages of assembly and then release the block and follow kinetically where the accumulated viral proteins move to-with respect to host compartment markers. No compartment of the Golgi can be unequivocally identified without a marker.

 The paper is not well described. For example the two virus mutants R7041 and R2641 are not well described. Why are they interesting? At the end of the discussion we learn that Us3 is a kinase. I could not find the interpretation for the acronym PNS, I eventually figured out it means peri nuclear space.

 In the background part they describe how gB/gH are reported as being essential for de-envelopment –the fusion step after budding into the nuclear envelope. When these proteins are missing one would expect enveloped virus to accumulate in the NE/ER. Yet they write- under this condition lots of capsids (without membrane) accumulate- and even infectious virus- so there must be an alternative exit route. As a question of logic the reader may ask: maybe the idea that these proteins are essential for de-envelopment is wrong?  

 Their description of the data on Us3 deletion mutants is not well explained.

 The close connection between the ER and the Golgi in uninfected cells has been seen by many EM studies, going back to Albert Claude 1970, Rambourg and many others. It is also not true as stated in the 1 ^st^ paragraph of their Discussion that the Golgi ‘disintegrates rapidly after improper fixation’-unless one forgets to add the aldehyde. They show a nice example of normal fixation in Fig 10B. I take issue with their claim that the 3D structure of the Golgi is poorly understood because of poor preservation. It is poorly understood because the organelle *is* complex and because one lacks systems to identify the different compartments in the same sections. I of course accept their claim that freeze substitution is the preferred option.

 The images from BFA treated cells are impressive but I’m not sure that I get a real take home message from these experiments. The Golgi fuses back with the ER; there is accumulation of budded virions in the ER. I cannot see a clear interpretation.

 The long paragraph in the beginning of page 10, dealing with the mutants is especially difficult to follow- e.g what does ‘accidentally proved’ mean?

 Among the many issues I have with their three models (Fig 11) I ask: how do they distinguish between budding into a Golgi compartment and wrapping? 

 Model 1 is the only one devoid of A. cytoplasmic capsids and B. ‘wrapping’ profiles. Doesn't this rule out this model?

 They poorly describe how they quantify virus particles in the different locations by EM. They must surely be referring the budding profiles to membrane length or compartment volume? (Page 4).

 The Conclusion part is also very difficult to relate to.

 Bottom of page 8 Dilates mis-spelt as ‘Delates’.

 Beginning of page 7 there is no word I know of in English – Exceptionalness’.

 It is a pity that such technically nice work is not supported by clarity of writing.

I have read this submission. I believe that I have an appropriate level of expertise to confirm that it is of an acceptable scientific standard, however I have significant reservations, as outlined above.

# Response

**Published:** 2018-02-16

**Authors:** Peter Wild

**Affiliations:** University of Zprich, Switzerland

Thank you for carefully reading the manuscript. You are right that the title may imply virus-induced formation of ER-to-Golgi transitions. However, it also may imply that viruses utilize existing transitions to transport virions from the PNS (abbreviation explained in introduction) into Golgi cisternae.

 Golgi structure does not differ in uninfected and infected cells except that in infected cells Golgi cisternae contain virions, which is clearly shown in Figs. 3 and 8D. Cells harvested or fixed e.g. 6 hpi (hours post inoculation) does not necessarily mean, that the cell under study is infected or that the infection occurred at the very moment of inoculation. Therefore, the Golgi complexes shown in Figs 3 and 8D are not (or not yet) involved in envelopment or transportation although capsids have been produced yet not obviously released from the nucleus.

 Immunolabelling does also not differ between uninfected and infected cells early in infection. However, the Golgi complex fragments about 16 hpi with wt HSV-1 as shown by Campadelli-Fiume et al., 1993. The reaction of the Golgi complex to infection with Us3 deletion mutant differs, which will be addressed in another article. The significance of Us3 will be included in introduction.

 To use markers for the different Golgi compartments is an interesting issue. As shown in Fig. 1, the cis-Golgi disappears by 16 hpi, whereas the trans-Golgi became fragmented. In contrast, both cis- and trans-Golgi enlarge after infection with the Us3 deletion mutant. The significance of Us3 on the Golgi complex will be addressed elsewhere.

 Markers of the viral envelope do not really help to locate the virions because they are present in the cytoplasm without virions, and even before the egress of virions starts (see e.g. de Oliveira et al., 2008).

 For simplicity we will show in Fig. 11 only the 2 pathways we propose: 1) the intraluminal pathway via ER-to-Golgi transitions, and 2) the pathway involving capsid release via impaired envelope (starting by pore dilation) followed by envelopment at Golgi membranes either by wrapping or budding. We have published enough data to support the direct nucleus to cytoplasm capsid translocation as well as to differentiate between wrapping and budding at Golgi membranes and vacuolar membranes.

 Numerous articles have been published using EM techniques and immunolabelling techniques on the light microscopic and EM level to try to support the idea of the envelopment – de-envelopment – re-envelopment pathway. Therefore, we do not repeat these experiments.

 The idea of de-envelopment (fusion of the viral envelope of virions in the PNS with the ONM releasing capsids and tegument into the cytoplasmic matrix) came from Stackpole (1968) to explain the presence of naked capsids in the cytoplasm. However, there is no correct prove that the process taking place at the ONM is fusion.

 Fact is that the processes taking place at the ONM is identical to that taking place at the INM showing all characteristics of budding as shown in detail earlier (Leuzinger et a., 2005).  Farnsworth et al., 2007 wanted to prove that the fusion proteins gB/gH are responsible for the fusion of the viral envelope with the ONM leading to accumulations of virions in the PNS. They show in their Fig. 2. -  now included in this paper as Fig. 12 - accumulation of virions in the PNS. “Accidentally” - or by mistake -, they overlooked about a dozen of virus interactions at the ONM. If these interactions were fusion virions would not have accumulated in the PNS. Consequently, 1) the process taking place at the ONM is budding not fusion, 2) gB/gH play other roles in the egress pathway than mediating fusion of the viral envelope with the ONM, and 3) the envelopment – de-envelopment – re-envelopment theory is incorrect, as shown earlier using Us3 deletion mutants.

 Us3 deletion mutants are infective although they almost completely accumulate in the PNS implying that Us3 null virions are not de-enveloped and re-enveloped. The same is true for the virions originating by budding at the INM after disintegration of the Golgi complex by BFA.

 We have revised the manuscript implementing your suggestion, and simplified the complicated paragraph dealing with the false interpretation that gB/gH promote fusion of the viral envelop with the ONM

 The “simple” take home massages are: Virions derive by budding at the INM membrane are infective. The process taking place at the ONM is budding, not fusion. The de-envelopment – re-envelopment theory is not valid.

# Response

**Published:** 2018-03-14

**Authors:** Peter Wild

**Affiliations:** University of Zprich, Switzerland

*Comment: The paper by Wild and colleagues investigates the question of how Herpes virus assembles, a story that has been under debate for decades. This family of DNA viruses assemble in the nucleus and thereafter opinions differ as to how the virus gets out of the cell. The bulk of the data are based on high pressure freezing and freeze substitution and are of exceptionally high quality.*

*However, I had a hard time trying to figure out what the point of the paper was. The title suggests a virus-induced ‘transition’ between ER and Golgi. I don't understand what they conclude about the ER-Golgi nor what it means for the virus. Virus-induced implies a difference between infected and uninfected cells.*

**Response: **Thank you for carefully reading the manuscript. You are right that the title may imply virus-induced formation of ER-to-Golgi transitions. However, it also may imply that viruses utilize existing transitions to transport virions from the PNS into Golgi cisternae. Virions are transported out of the PNS into ER cisternae. ER-Golgi transitions may thus be used to transport virions directly into Golgi cisternae. There are evidences that this idea is likely to be correct.

**

*The only image of an uninfected cell is the IF image in Fig 1. I don't see much difference in the pattern of GM-130 labeling, a marker of early Golgi compartments. They show no EM images of uninfected cell Golgi.*

**Response: **Golgi structure does not differ in uninfected and infected cells except that in infected cells Golgi cisternae contain virions, which is clearly shown in Figs. 3 and 8D. Cells harvested or fixed e.g. 6 hpi (hours post inoculation) does not necessarily mean, that the cell under study is infected or that the infection occurred at the very moment of inoculation. Therefore, the Golgi complexes shown in Figs 3 and 8D are not (or not yet) involved in envelopment or transportation although capsids might have been produced yet not obviously released from the nucleus and transported into the Golgi complex.

Immunolabelling does also not differ between uninfected and infected cells early in infection. However, the Golgi complex fragments about 16 hpi with wt HSV-1 as shown by Campadelli-Fiume et al., 1993. The Golgi complex enlarges after infection with Us3 deletion mutant, which will be addressed in another article. The revised Fig. shows the cis and trans Golgi after infection with HSV-1 and the Us3 deletion mutant.

*I had a serious problem also trying to understand how they could distinguish between the three models shown in Fig 11. I could not follow their logic.*

**Response: **We can only distinguish between two pathways: 1) Via budding at nuclear membranes followed by intraluminal transportation, and 2) capsid release via impaired nuclear envelope (that starts by pore dilation) followed by envelopment at Golgi membranes or membranes of the ER. This can be easily done based on the diverse phenotypes. For details see Leuzinger 2005, Wild 2002, 2005, 2009

Pathway 3 (which is omitted in the revised manuscript) is not our idea. The idea of de-envelopment (fusion of the viral envelope of virions in the PNS with the ONM releasing capsids and tegument into the cytoplasmic matrix) came from Stackpole (1968) to explain the presence of naked capsids in the cytoplasm. However, there is no correct prove that the process taking place at the ONM is fusion.

*.*

*In my opinion to do this in a satisfactory was they would need to use host cell markers for the different Golgi compartments with use of some viral membrane markers.*

**Response: **Where is no need to distinguish between Golgi compartments in this context. The question is: can virions be transported from the ER into the Golgi to what compartment ever. However, to use markers for the different Golgi compartments is an interesting issue considering Golgi fragmentation as shown in the revised Fig 1. (work is in progress). Markers of the viral envelope do not really help to locate the virions because they are present in the cytoplasm without virions, and even before the egress of virions starts (see e.g. de Oliveira et al., 2008).

*To be able to give directionality to their static images they would also need e.g an inducible system whereby they could block some stages of assembly and then release the block and follow kinetically where the accumulated viral proteins move to-with respect to host compartment markers.*

**Response: **As stated above, viral proteins are not useful to track virus particles. This is shown in the revised Fig. 1 showing the viral protein gB throughout the cytoplasm. To localize the 3 viral compartments (capsid, tegument, envelope) we constructed a triple coloured virus (Oliveira et al.2008).

*No compartment of the Golgi can be unequivocally identified without a marker.*

**Response:** In an image as shown in Fig. 9A one can easily identify the cis-and trans-face. **

*The paper is not well described. For example the two virus mutants R7041 and R2641 are not well described. Why are they interesting? At the end of the discussion we learn that Us3 is a kinase.*

**Response:** The knowledge of the significance of Us3 is not important considering ER-to-Golgi transitions. One important aspect is that Us3 downregulates phospholipid biosynthesis leading to enlargement of the Golgi complex if Us3 is absent as shown in the revised Fig. 1. The significance of Us3 on Golgi assembly early in infection is under study. R2641 is the repair mutant and does not need any explanation. The significance of Us3 is described in introduction of the revised manuscript.

*I could not find the interpretation for the acronym PNS, I eventually figured out it means peri nuclear space.*

**Response:** It was and still is in “introduction”

*In the background part they describe how gB/gH are reported as being essential for de-envelopment –the fusion step after budding into the nuclear envelope. When these proteins are missing one would expect enveloped virus to accumulate in the NE/ER. Yet they write- under this condition lots of capsids (without membrane) accumulate- and even infectious virus- so there must be an alternative exit route. As a question of logic the reader may ask: maybe the idea that these proteins are essential for de-envelopment is wrong?*

**Response: **Yes, this idea is wrong. The process taking place at the outer nuclear membrane shows all characteristics of budding, none of fusion. It takes place in the absence of the fusion proteins gB and gH leading to accumulation of virions in the PNS. This is a clear indication for budding even for those who are not familiar with the fundamentals of membrane bound transportation. By the way, to distinguish between budding and fusion is as easy as to distinguish between the right and the left shoe.

*Their description of the data on Us3 deletion mutants is not well explained.*

**Response: **The only things we will show is ER-to-Golgi transitions, which we found often in cells infected with the Us3 deletion mutant. This has possibly something to do with the enlargement of the Golgi in the absence of Us3.

*The close connection between the ER and the Golgi in uninfected cells has been seen by many EM studies, going back to Albert Claude 1970, Rambourg and many others.*

**Response:** In my opinion, the question is not whether connections between ER and Golgi exist. The question is whether these connections are used for transport of cargo from the ER into Golgi cisternae. Considering the enormous amount of proteins produced and secreted in the exocrine pancreas (1.2 litter per day in humans) it seems to me likely that such a route exists.

*It is also not true as stated in the 1 ^st^ paragraph of their Discussion that the Golgi ‘disintegrates rapidly after improper fixation’-unless one forgets to add the aldehyde.*

**Response:** This is certainly true. One can destroy the Golgi complex e.g. by using an inappropriate buffer during fixation. I know that you did excellent studies on Golgi structure and function. Unfortunately, there are too many papers published showing inadequate preservation of Golgi structure, including papers dealing with the egress of herpes viruses.

*They show a nice example of normal fixation in Fig 10B. I take issue with their claim that the 3D structure of the Golgi is poorly understood because of poor preservation. It is poorly understood because the organelle is complex and because one lacks systems to identify the different compartments in the same sections. I of course accept their claim that freeze substitution is the preferred option.*

**Response: **The complicated 3D structure plays certainly an important role that the Golgi is poorly understood.

*The images from BFA treated cells are impressive but I’m not sure that I get a real take home message from these experiments. The Golgi fuses back with the ER; there is accumulation of budded virions in the ER. I cannot see a clear interpretation.*

**Response: **The accumulation of virions is the result of impaired transportation out of the PNS-ER compartment. Since the process taking place at the ONM is budding not fusion it is reasonable to assume that the intraluminal transport to the Golgi complex is impaired as was shown also for proteins. Furthermore, it is also an additional indication that the process at the ONM is not fusion because it is unlikely that disintegration of the Golgi would have an impact on the fusion process.

Us3 deletion mutants are infective although they almost completely accumulate in the PNS implying that Us3 null virions are not de-enveloped and re-enveloped. The same is true for the virions originating by budding at the INM, ONM and ER membranes after disintegration of the Golgi complex by BFA.

The real take home message is that the theory of de-re-envelopment is essential for production of infectious progeny virus is not valid.

*The long paragraph in the beginning of page 10, dealing with the mutants is especially difficult to follow- e.g what does ‘accidentally proved’ mean?*

**Response:** Accidentally means the following: Farnsworth et al., 2007 wanted to prove that the fusion proteins gB/gH are responsible for the fusion of the viral envelope with the ONM leading to accumulations of virions in the PNS. They show in their Fig. 2. -  now included in this paper as Fig. 12 - accumulation of virions in the PNS. “Accidentally” - or by mistake -, they overlooked about a dozen of virus interactions at the ONM and ER. If these interactions were fusion virions would not have accumulated in the PNS. Consequently, 1) the process taking place at the ONM is budding not fusion, 2) gB/gH play other roles (if any?) in the egress pathway than mediating fusion of the viral envelope with the ONM, and 3) the envelopment – de-envelopment – re-envelopment theory is incorrect, as shown earlier using Us3 deletion mutants (see above).

*Among the many issues I have with their three models (Fig 11) I ask: how do they distinguish between budding into a Golgi compartment and wrapping?*

**

**Response: **Capsids can bud into vacuoles and cisternae of any size even if they contain already virions. During wrapping, the viral envelope and the vacuole are concomitantly formed. The result is a concentric vacuole containing a single virion. The space between virion and vacuolar membrane is filled by dense material that probably had driven the process. For details see Leuzinger et al. 2005 (JV), and Wild et al. 2002 (Micron).

*Model 1 is the only one devoid of A. cytoplasmic capsids and B. ‘wrapping’ profiles. Doesn't this rule out this model?*

**Response:** Model 1 is the intraluminal pathway: Capsids arise by budding at the INM, ONM or ER membranes and are intraluminally transported. The destination is considered likely to be Golgi cisternae via ER-Golgi transitions.

In model 2, capsids gain access to the cytoplasmic matrix via impaired nuclear envelope that starts by dilation of nuclear pores. See Leuzinger 2005, Wild 2005, 2009.

*They poorly describe how they quantify virus particles in the different locations by EM. They must surely be referring the budding profiles to membrane length or compartment volume? (Page 4).*

**Response: **We expressed the number of virus particles per cellular profile. I know that this is not very appropriate. However, this was done by many other authors addressing herpes virus envelopment. Since it is obvious that virions accumulate in the PNS we consider the data to be sufficiently accurate. In the study about the significance of Us3 on the biosynthesis of phospholipids, we expressed the morphometric data per mean nuclear and cell volume, respectively (Wild et al., 2012).

*The Conclusion part is also very difficult to relate to.*

*Bottom of page 8 Dilates mis-spelt as ‘Delates’.*

*Beginning of page 7 there is no word I know of in English – Exceptionalness’.*

*It is a pity that such technically nice work is not supported by clarity of writing.*

**

**Response:** It is difficult to write an article about a process that was misinterpreted for decades ignoring the fundamentals of membrane bound transportation, and that was pushed to an untouchable dogma.

We have revised the manuscript implementing your suggestion, and simplified the complicated paragraph dealing with the false interpretation that gB/gH promote fusion of the viral envelop with the ONM. We also made clear in the conclusions that the significance of many viral proteins must be re-investigated in the light of correct interpretation of viral-membrane interactions.

# Peer review: Referee response for version 1

**DOI:** 10.5256/f1000research.13264.r26712

**Published:** 2017-10-10

**Authors:** Charles Grose

**Affiliations:** 1 Virology Laboratory, Children's Hospital, University of Iowa, Iowa City, IA, 52242, USA

Professor Wild and his collaborators are well known for their expertise in electron microscopy. I also note that three other scientists who developed cryo-TEM won the Nobel Prize this year. The SEM and TEM images in this manuscript are excellent. I enjoyed reviewing their data. I have heard Professor Wild present his results at conferences. However, I can say upfront that most recent herpesvirus data appear to support pathway #3 in Figure 11, which is not the pathway preferred by the Wild lab. For example, the authors do not consider that both the Elliott HSV lab and the C. Grose VZV lab have found evidence for Rab11 on the vesicles that transport enveloped virus. If the above two labs are correct, the pathway #1 in Figure 11 cannot be completely correct because the drawings do not include the endocytic pathway. Thus, the experiment requested in Comment 6 needs to be performed to resolve this question. Overall, the suggestion is made that the Wild lab take a look at some of the VZV articles and include them in their discussion, even if the Wild lab disagree with the conclusions of the VZV lab.

  

1. Figure 1. Confocal microscopy of Vero cells. The text states that both WT and mutant virus were tested but only one virus is shown in Figure 1. Which virus is shown? If only WT virus is shown, may wish to add confocal with mutant virus.
2. Figures 4 and 5. Virions in ER cisternae. The C. Grose lab has published several articles about VZV encapsidation, VZV capsid transport from nucleus to cytoplasm and subsequent envelopment.  These articles should be reviewed and cited by Wild *et al.*  First of all, Wild should show us whether he finds viral particles between the INM and the ONM. For VZV, Harson and Grose (1995) have clearly shown that capsids with a thin envelope can be found between the INM and the ONM. See Harson and Grose, Figure 5, panels C, D and E. Note increase in diameter of particle between panels C and E. Panel F is important because panel F shows a thin-enveloped particle still within the ER cisternae and clearly beyond the ONM. Thus, the VZV micrograph agrees with the data from Wild about “Virions are within ER cisternae.”
3. Data not shown by Wild lab. Harson and Grose have shown that VZV capsids without a thin envelope can be found in the cytoplasm. The capsids do not appear to be within any Golgi derived compartment. See Figure 6 panel A, (Harson & Grose, 1995). This micrograph provides evidence against the Wild hypothesis that herpesviruses are always retained within compartments derived from the ER or Golgi. There is one micrograph in the Wild manuscript that appears to resemble the VZV micrograph. See Wild manuscript Figure 9B, which shows a BoHV capsid (without a thin envelope) in the cytoplasm adjacent to a Golgi (or ER/Golgi) membrane. This single BoHV micrograph would seem to invalidate the Wild hypothesis that BoHV always is transported within ER or Golgi compartments?
4. Differences between viral particles in ER cisternae and viral particles in vesicles near the outer cell membrane. As noted above, data from the Grose lab agree with data from the Wild lab that viral particles are found in ER cisternae. But the Grose lab finds differences between particles in ER cisternae and particles in vesicles near the outer cell membrane. ER cistern are irregularly shaped. Particles within ER cisternae have a thin envelope without an obvious electron dense outer coat (presumably the viral glycoproteins). Vesicles containing viral particles near the outer cell membrane often are circular with a single outer membrane. See Figure 8 panel A in Harson & Grose (1995), for a clear VZV example. The vesicle contains 4 particles, 2 of which are perfectly enveloped, with clearly defined capsid, tegument and envelope with electron-dense outer coat. These particles are slightly larger in diameter than the thin-enveloped particles seen in the irregular ER cisternae. The VZ virions in the circular vesicles are similar to the BoHV virions shown by Wild in his Figure 9 panels D and E. In other words, there clearly are two types of enveloped particles: particles with thin-envelope and particles with a thicker electron-dense envelope.
5. Virions are within Golgi cistern? Wild discusses viral particles with spikes and viral particles without obvious spikes. Spikes presumably represent mature glycoproteins. One possibility that Wild has not considered is that viral particles may be routed in different pathways. Some viral particles may enter the large Golgi-like cisternae. Other viral particles may be wrapped in individual vesicles. Why does there need to be a single pathway for all viral particles?
6. Lack of data about endocytic or autophagy pathways. There are now HSV and VZV papers that provide evidence for involvement of the endocytic and/or autophagy pathways in viral egress. The Elliott lab has shown that both Rab5 and Rab11 are involved in the final envelopment process for HSV. Further, they did not find co-localization of capsids with the TGN marker TGN46. See Hollinshead *et al.* 2012. The C. Grose lab confirmed that a fraction of purified VZ virions were positive for the Rab11 protein, presumably because the purified virions retained remnants of a Rab11-positive vesicle. See VZV article by Buckingham *et al*, 2016. The C. Grose lab also found the LC3-II protein in the same purified virion fraction and hypothesized that the virions were housed in amphisome-like vesicles, which contain both Rab11 and LC3-II proteins in their membrane. The fact that a HSV lab and a VZV lab both found Rab11 in the virion greatly strengthens the observation. These data are presented because they suggested that the Wild lab cannot determine the origin of the vesicles containing either HSV or BoHV virions by using only TEM and SEM. The Wild lab will need to use either immuno-TEM or alternatively isolate the HSV and BoHV particles, then examine them for proteins such as Rab11 and LC3-II by immunoblotting.
7. Figure 11. Schematic drawings. If the Elliott lab and the Grose lab are correct that purified HSV or VZV contain Rab11 protein, then schematic pathway #1 in Figure 11 cannot be correct. Also, if the Elliott lab and the Grose lab are correct, then secondary envelopment must involve an intersection of the virus with a pathway outside of the ER/Golgi pathway. Strongly suggest that the Wild lab investigate whether any of the vacuoles that contain either HSV or BoHV are positive for the Rab11 protein. Electron microscopy alone is not sufficient.
8. Figure 7. Virus titers. cell by endocytosis. The titers at 8hpi probably represent viral particles formed after some actual HSV glycoprotThe data about the different titers with and without BFA may need re-interpretation. The titer is much lower when BFA was added at 5hpi. Most of these particles would be capsids and capsids with thin-envelopes. A titer of 1000 PFU/ml seems reasonable for capsids, which could enter a ein biosynthesis. Some VZV glycoproteins undergo their biosynthesis within 2 hours. See article from Grose lab by Yao *et al*, 1993. Presumably HSV glycoproteins are synthesized at the same rate. Therefore, addition of BFA at 8hpi would not prevent formation of substantial amounts of HSV glycoproteins, which would allow envelopment of viral particles, even by 8 hpi. In other words, I do not think that the BFA experiments add much weight to the arguments about which pathway HSV takes
9. Bovine herpesvirus data. I do find inclusion of the BoHV micrographs to be informative. The BoHV data appear to resemble the VZV data more closely than the HSV data.  Remember that BoHV is a member of the varicellovirus subfamily.
10. Schematic pathway for VZV trafficking. Please take the time to look over the VZV pathway illustrated in Figure 8, in article by Buckingham *et al*, 2016. It resembles pathway #3 by the Wild group.

I have read this submission. I believe that I have an appropriate level of expertise to confirm that it is of an acceptable scientific standard, however I have significant reservations, as outlined above.

## References

[1] HarsonRGroseC: Egress of varicella-zoster virus from the melanoma cell: a tropism for the melanocyte. *J Virol*.1995;69(8) :4994-5010 760907010.1128/jvi.69.8.4994-5010.1995PMC189316

[2] HollinsheadMJohnsHLSayersCLGonzalez-LopezCSmithGLElliottG: Endocytic tubules regulated by Rab GTPases 5 and 11 are used for envelopment of herpes simplex virus. *EMBO J*.2012;31(21) : 10.1038/emboj.2012.262 4204-20 10.1038/emboj.2012.262 22990238PMC3492727

[3] BuckinghamEMJarosinskiKWJacksonWCarpenterJEGroseC: Exocytosis of Varicella-Zoster Virus Virions Involves a Convergence of Endosomal and Autophagy Pathways. *J Virol*.2016;90(19) : 10.1128/JVI.00915-16 8673-85 10.1128/JVI.00915-16 27440906PMC5021422

[4] YaoZJacksonWForghaniBGroseC: Varicella-zoster virus glycoprotein gpI/gpIV receptor: expression, complex formation, and antigenicity within the vaccinia virus-T7 RNA polymerase transfection system. *J Virol*.1993;67(1) :305-14 838007810.1128/jvi.67.1.305-314.1993PMC237364

# Response

**Published:** 2018-03-14

**Authors:** Peter Wild

**Affiliations:** University of Zprich, Switzerland

*Professor Wild and his collaborators are well known for their expertise in electron microscopy. I also note that three other scientists who developed cryo-TEM won the Nobel Prize this year. The SEM and TEM images in this manuscript are excellent. I enjoyed reviewing their data. I have heard Professor Wild present his results at conferences. However, I can say upfront that most recent herpesvirus data appear to support pathway #3 in Figure 11, which is not the pathway preferred by the Wild lab. For example, the authors do not consider that both the Elliott HSV lab and the C. Grose VZV lab have found evidence for Rab11 on the vesicles that transport enveloped virus. If the above two labs are correct, the pathway #1 in Figure 11 cannot be completely correct because the drawings do not include the endocytic pathway. Thus, the experiment requested in Comment 6 needs to be performed to resolve this question. Overall, the suggestion is made that the Wild lab take a look at some of the VZV articles and include them in their discussion, even if the Wild lab disagree with the conclusions of the VZV lab.*

**Response: **Thank you for the compliment. It is great honour to have become influenced by Jacques Dubochet, one of the Nobel prize winners on cryo-TEM. I am also very grateful for the impact of another Nobel prize winner, George Palade, whose laureate speech entitled “Variation on a common theme in the secretory pathway” published in Science,1975, is still a must to be read by people working in this field, including herpes virologists.

The intention of this work is to demonstrate connectivity between the PNS and the Golgi complex that may be used as a direct transport pathway for virions derived by budding at nuclear membranes. Budding at nuclear membranes followed by intraluminal transport is referred to as pathway 1. 

That does not mean that our lab prefers pathway 1. We also showed that capsids gain access to the cytoplasmic matrix via impaired nuclear pores and impaired nuclear envelope, respectively. These capsids follow envelopment pathway 2. In this pathway, the endocytic pathway will be included (we did not because, we shall discuss it in detail in another manuscript). Pathway 3 is obsolete, because the process at the outer nuclear membrane is budding not fusion as accidentally proved by Farnsworth et all, 2007, overlooking the process taking place at the ONM (see Fig.12).

**

*Figure 1. Confocal microscopy of Vero cells. The text states that both WT and mutant virus were tested but only one virus is shown in Figure 1. Which virus is shown? If only WT virus is shown, may wish to add confocal with mutant virus.*

**Response: **The location of the Golgi complex depends on the cell, not on the virus. Nevertheless, we included micrographs of Golgi labelled with antibodies against the cis and the trans face in cells infected with HSV-1 and the Us3 deletion mutant thereof. Fragmentation of the Golgi complex depends on the virus. E.g. in Us3 deletion mutant infected cells, the Golgi complex enlarges, A manuscript addressing the significance of Us3 on the Golgi complex is in progress.

*Figures 4 and 5. Virions in ER cisternae. The C. Grose lab has published several articles about VZV encapsidation, VZV capsid transport from nucleus to cytoplasm and subsequent envelopment.  These articles should be reviewed and cited by Wild et al.  First of all, Wild should show us whether he finds viral particles between the INM and the ONM.*

**Response: **We have shown many times virions between the INM an ONM as well as in adjacent cisternae. However, we never found virions with a thin envelope. This might be related to the different fixation and preparation procedure.

*For VZV, Harson and Grose (1995) have clearly shown that capsids with a thin envelope can be found between the INM and the ONM. See Harson and Grose, Figure 5, panels C, D and E. Note increase in diameter of particle between panels C and E. Panel F is important because panel F shows a thin-enveloped particle still within the ER cisternae and clearly beyond the ONM. Thus, the VZV micrograph agrees with the data from Wild about “Virions are within ER cisternae.”*

*Data not shown by Wild lab. Harson and Grose have shown that VZV capsids without a thin envelope can be found in the cytoplasm.*

**Response:** We have shown capsids in the cytoplasm in this manuscript (Figs.4B and 9B) and plotted the total number of capsids in the cytoplasm in Fig. 6. We also showed capsids in many articles published earlier, e.g. Wild et al. 2005 (impairment of nuclear pores) or Leuzinger et al.2005 (The dual pathway). In these articles the difference between budding at Golgi membranes and wrapping by Golgi membranes is clearly shown.

*The capsids do not appear to be within any Golgi derived compartment. See Figure 6 panel A, (Harson & Grose, 1995). This micrograph provides evidence against the Wild hypothesis that herpesviruses are always retained within compartments derived from the ER or Golgi. There is one micrograph in the Wild manuscript that appears to resemble the VZV micrograph. See Wild manuscript Figure 9B, which shows a BoHV capsid (without a thin envelope) in the cytoplasm adjacent to a Golgi (or ER/Golgi) membrane. This single BoHV micrograph would seem to invalidate the Wild hypothesis that BoHV always is transported within ER or Golgi compartments?*

**Response: **As discussed above, we postulate 2 pathways: 1) Budding at nuclear membranes followed by intraluminal transportation, 2) Release via impairs nuclear envelope followed by budding into Golgi (and other) membranes, or by wrapping (see Leuzinger et al, 2005, Wild et.al. 2005 and 2009). In introduction, we inserted the pathway via vesicle formation. We have also observed some formation of vesicles late in infection.

*Differences between viral particles in ER cisternae and viral particles in vesicles near the outer cell membrane. As noted above, data from the Grose lab agree with data from the Wild lab that viral particles are found in ER cisternae. But the Grose lab finds differences between particles in ER cisternae and particles in vesicles near the outer cell membrane. ER cistern are irregularly shaped. Particles within ER cisternae have a thin envelope without an obvious electron dense outer coat (presumably the viral glycoproteins). Vesicles containing viral particles near the outer cell membrane often are circular with a single outer membrane. See Figure 8 panel A in Harson & Grose (1995), for a clear VZV example. The vesicle contains 4 particles, 2 of which are perfectly enveloped, with clearly defined capsid, tegument and envelope with electron-dense outer coat. These particles are slightly larger in diameter than the thin-enveloped particles seen in the irregular ER cisternae. The VZ virions in the circular vesicles are similar to the BoHV virions shown by Wild in his Figure 9 panels D and E. In other words, there clearly are two types of enveloped particles: particles with thin-envelope and particles with a thicker electron-dense envelope.*

**Response: **Using freezing and substitution techniques we did not find differences in the viral envelope of virions in the PNS and ER. The virus particles in Harson at al. differ most probably because of preservation quality (in panel 8A much of the substances are lost) and of the section plane.

*Virions are within Golgi cistern? Wild discusses viral particles with spikes and viral particles without obvious spikes. Spikes presumably represent mature glycoproteins. One possibility that Wild has not considered is that viral particles may be routed in different pathways. Some viral particles may enter the large Golgi-like cisternae. Other viral particles may be wrapped in individual vesicles. Why does there need to be a single pathway for all viral particles?*

**Response: **As stated above, we propose two diverse pathways indicated in Fig, xx by 1 and 2. However, we do not agree with the postulation, that de-envelopment followed by re-envelopment is essential for production of infectious progeny virus because Us3 deletion mutants are fully effective although 98% of these virions accumulate in the PNS and adjacent ER. 

*Lack of data about endocytic or autophagy pathways. There are now HSV and VZV papers that provide evidence for involvement of the endocytic and/or autophagy pathways in viral egress. The Elliott lab has shown that both Rab5 and Rab11 are involved in the final envelopment process for HSV. Further, they did not find co-localization of capsids with the TGN marker TGN46. See Hollinshead et al. 2012. The C. Grose lab confirmed that a fraction of purified VZ virions were positive for the Rab11 protein, presumably because the purified virions retained remnants of a Rab11-positive vesicle. See VZV article by Buckingham et al, 2016. The C. Grose lab also found the LC3-II protein in the same purified virion fraction and hypothesized that the virions were housed in amphisome-like vesicles, which contain both Rab11 and LC3-II proteins in their membrane. The fact that a HSV lab and a VZV lab both found Rab11 in the virion greatly strengthens the observation. These data are presented because they suggested that the Wild lab cannot determine the origin of the vesicles containing either HSV or BoHV virions by using only TEM and SEM. The Wild lab will need to use either immuno-TEM or alternatively isolate the HSV and BoHV partic*les, then examine them for proteins such as Rab11 and LC3-II by immunoblotting.   

    

**Response:** The intention of this manuscript is to discover a possible intraluminal transportation route from the PNS into Golgi cisternae. No more no less. By no means does it question the existence of an “endocytic” pathway. Therefore, there is no need to repeat experiments other groups have done as cited for HSV-1 in introduction (Albecka et al. 2016, Holinstead et al., 2012). We will describe in detail this pathway in another manuscript. Considering membrane recycling, it would be a great surprise if an “endocytic” pathway would not exist. However, I do not think that capsids bud at endosomes per se. Possibly, they rather bud at membrane compartments that contain recycled (endosomal) membrane components.

**

*Figure 11. Schematic drawings. If the Elliott lab and the Grose lab are correct that purified HSV or VZV contain Rab11 protein, then schematic pathway #1 in Figure 11 cannot be correct. Also, if the Elliott lab and the Grose lab are correct, then secondary envelopment must involve an intersection of the virus with a pathway outside of the ER/Golgi pathway. Strongly suggest that the Wild lab investigate whether any of the vacuoles that contain either HSV or BoHV are positive for the Rab11 protein. Electron microscopy alone is not sufficient.*

**Response: **The endocytic pathway will be included in Fig. 11. For simplicity, we removed pathway 3 because we did not find correct proves it. Instead, we introduced Figure 2 of Farnsworth et al., 2007 showing transport of capsids across the ONM and ER membranes in the absence of the fusion proteins gB/gH and accumulation of virions in the PNS-ER compartment.

*Figure 7. Virus titers. cell by endocytosis. The titers at 8hpi probably represent viral particles formed after some actual HSV glycoprotThe data about the different titers with and without BFA may need re-interpretation. The titer is much lower when BFA was added at 5hpi. Most of these particles would be capsids and capsids with thin-envelopes. A titer of 1000 PFU/ml seems reasonable for capsids, which could enter a ein biosynthesis. Some VZV glycoproteins undergo their biosynthesis within 2 hours. See article from Grose lab by Yao et al, 1993. Presumably HSV glycoproteins are synthesized at the same rate. Therefore, addition of BFA at 8hpi would not prevent formation of substantial amounts of HSV glycoproteins, which would allow envelopment of viral particles, even by 8 hpi. In other words, I do not think that the BFA experiments add much weight to the arguments about which pathway HSV takes*

**Response:** Glycoprotein of HSV-1start to be synthesised prior to 4 hpi and are localized at the Golgi complex (unpublished data) which is in agreement with the biosynthesis of glycoproteins in VZV. In the work of de Oliviera et al., 2008, gH was found in the cytoplasm and at the nuclear periphery at 4hpi. The proteins at nuclear membranes are used for envelopment after disintegration of the Golgi complex. The only way capsids can be enveloped after disintegration of the Golgi complex is by budding at nuclear and ER membranes. An important question is: where the capsids get their envelope after fragmentation of the Golgi complex in HSV-1 infection (Campadelli-Fiume et al., 1993)?

*Bovine herpesvirus data. I do find inclusion of the BoHV micrographs to be informative. The BoHV data appear to resemble the VZV data more closely than the HSV data.  Remember that BoHV is a member of the varicellovirus subfamily.*

**Response: **The images of BoHV nicely show connectivity between PNS and Golgi via RER as well as the juxtanuclear position of the Golgi complex. Therefore, it is an important image to understand the relation between Golgi, ER, and PNS. ER-to Golgi transitions have been shown in many cells and are not induced by herpes viruses (see also comments of Gareth Griffiths). 

*Schematic pathway for VZV trafficking. Please take the time to look over the VZV pathway illustrated in Figure 8, in article by Buckingham et al, 2016. It resembles pathway #3 by the Wild group.*

**Response: **Pathway 3 was omitted in Fig. 11 because it is not our idea, and because the process at the ONM is not fusion (de-envelopment) as shown using a mutant lacking the fusion proteins gB/gH that results in accumulation of virions in the PNS (see Fig.12). By the way, to distinguish between budding and fusion is as easy as to distinguish between the right and left shoe.
